# Supplementary material for: Photobiomodulation for pain management during placement of the copper T 380 intrauterine device: Protocol for a randomized, double-blind controlled trial
Source: PLoS One. 2026 May 28;21(5):e0349031. doi: 10.1371/journal.pone.0349031 (PMC13218537; doi:10.1371/journal.pone.0349031)
Supplement: S2 File — This is the S2 File legend; there is no legend. (DOCX) [file pone.0349031.s002.docx]

**UNIVERSIDADE NOVE DE JULHO**

**PROGRAMA DE PÓS-GRADUAÇÃO EM BIOFOTÔNICA APLICADA ÀS CIÊNCIAS DA SAÚDE**

**Anna Carolina** **Nunes Ferraz**

**EFEITO DA FOTOBIOMODULAÇÃO NA REDUÇÃO DA PERCEPÇÃO DE DOR DURANTE A INSERÇÃO DE DIU DE COBRE T 380 PARA A CONTRACEPÇÃO:** **ESTUDO CLÍNICO CONTROLADO RANDOMIZADO**

**São Paulo**

**2024**

**UNIVERSIDADE NOVE DE JULHO**

**PROGRAMA DE PÓS-GRADUAÇÃO EM BIOFOTÔNICA APLICADA ÀS CIÊNCIAS DA SAÚDE**

**Anna Carolina Nunes Ferraz**

**EFEITO DA FOTOBIOMODULAÇÃO NA REDUÇÃO DA PERCEPÇÃO DE DOR DURANTE A INSERÇÃO DE DIU DE COBRE T 380 PARA A CONTRACEPÇÃO:** **ESTUDO CLÍNICO CONTROLADO RANDOMIZADO**

|  | Projeto de pesquisa apresentado ao Comitê de Ética em pesquisa do Conjunto Hospitalar do Mandaqui.  Orientadora: Profª Drª Anna Carolina Ratto Tempestini Horliana.  Co-Orientadora: Kristianne Porta Santos Fernandes |
| --- | --- |

**São Paulo**

**2024**

**Resumo**

A gravidez não planejada afeta até 65% das mulheres em algumas regiões do Brasil, elevando os riscos de abortos inseguros e contribuindo para a mortalidade materna. O DIU de cobre é uma alternativa contraceptiva eficaz e de longa duração, mas seu uso ainda é limitado no Brasil, abrangendo apenas 4,4% das mulheres em idade reprodutiva. Uma das principais barreiras é a dor associada à sua inserção, que gera medo e baixa adesão ao método. Como a dor pode ser de origem visceral ou somática, abordagens tradicionais como anti-inflamatórios e anestésicos mostram resultados inconclusivos na redução desse desconforto. A fotobiomodulação (FBM) promove efeitos anti-inflamatórios e analgésicos, apresentando resultados positivos no controle da dor pélvica em outros contextos clínicos, como o trabalho de parto. O objetivo deste estudo é avaliar a eficácia da FBM como método analgésico preemptivo na inserção do DIU de cobre T 380. Será realizado um ensaio clínico randomizado, duplo-cego, com 72 participantes distribuídas aleatoriamente entre grupo experimental (n=36) - FBM ativa e grupo controle (n=36) - simulação da FBM. As pacientes seguirão o protocolo de inserção do DIU conforme diretrizes do Ministério da Saúde. A dor será avaliada em diferentes momentos utilizando a Escala Visual Analógica (EVA) durante as fases de inserção (Pozzi, Histereometria e a inserção) 5 e 15 minutos, 24 e 48 horas após a inserção do DIU. Além disso, serão investigados o uso de analgésicos e qualidade de vida (WHOQOL-100) no período de 48 horas, níveis de ansiedade (GAD-7), satisfação com o procedimento logo após a inserção (15 minutos) e efeitos adversos e colaterais em um período de 48 horas. Também será avaliada a duração da dor em horas desde o momento da inserção do DIU até o seu término. E o índice de sucesso do procedimento. A análise estatística será realizada no software SPSS versão 24.0, com nível de significância de 5% (p < 0,05). A normalidade dos dados será avaliada pelo teste de Shapiro-Wilk. Testes t de Student ou Mann-Whitney serão usados para variáveis contínuas, e o teste qui-quadrado ou exato de Fisher para variáveis categóricas. A dor será analisada pelo teste de Friedman e a regressão logística avaliará associações entre grupos e efeitos adversos. A análise estatística será realizada com um nível de significância de 5%. A normalidade dos dados será avaliada pelo teste de Shapiro-Wilk. Para a análise da dor (EVA) e de variáveis como ansiedade e qualidade de vida, será aplicado o teste de Friedman. O uso de analgésicos será avaliado por ANOVA para medidas repetidas. Os efeitos adversos serão analisados por regressão logística. O tempo para cessar o desconforto abdominal será estimado por meio da análise de Kaplan-Meier, e o sucesso de inserção do DIU será comparado utilizando o teste qui-quadrado.

**Palavras chave:** métodos contraceptivos, dispositivo intrauterino (diu), controle da dor, terapia por fotobiomodulação, ensaio clínico randomizado

**Abstract**

Unplanned pregnancy affects up to 65% of women in some regions of Brazil, increasing the risks of unsafe abortions and contributing to maternal mortality. The copper IUD is an effective and long-lasting contraceptive option, but its use is still limited in Brazil, covering only 4.4% of women of reproductive age. One of the main barriers is the pain associated with its insertion, which leads to fear and low adherence to the method. Since pain can be of visceral or somatic origin, traditional approaches such as anti-inflammatories and anesthetics have shown inconclusive results in reducing this discomfort. Photobiomodulation (PBM) has anti-inflammatory and analgesic effects, and has shown positive results in managing pelvic pain in other clinical contexts, such as labor. The objective of this study is to evaluate the efficacy of PBM as a preemptive analgesic method during the insertion of the T 380 copper IUD. A randomized, double-blind clinical trial will be conducted with 72 participants randomly allocated into an experimental group (n=36) – active PBM and a control group (n=36) – PBM simulation. Patients will follow the IUD insertion protocol according to the Ministry of Health guidelines. Pain will be assessed at different time points using the Visual Analog Scale (VAS) during the insertion phases (Pozzi, hysterometry, and IUD insertion), at 5 and 15 minutes, and at 24 and 48 hours after IUD insertion. Additionally, analgesic use and quality of life (WHOQOL-100) will be assessed over a 48-hour period, along with anxiety levels (GAD-7), satisfaction with the procedure immediately after insertion (15 minutes), and adverse and side effects within 48 hours. The duration of pain in hours from the moment of IUD insertion until its resolution will also be evaluated, as well as the success rate of the procedure. Statistical analysis will be performed using SPSS software version 24.0, with a significance level of 5% (p < 0.05). Data normality will be assessed using the Shapiro-Wilk test. Student’s t-test or the Mann-Whitney test will be used for continuous variables, while the chi-square test or Fisher’s exact test will be applied for categorical variables. Pain will be analyzed using the Friedman test, and logistic regression will evaluate associations between groups and adverse effects. Statistical analysis will be performed with a significance level of 5%. Data normality will be assessed using the Shapiro-Wilk test. For pain analysis (VAS) and variables such as anxiety and quality of life, the Friedman test will be applied. Analgesic use will be evaluated using repeated measures ANOVA. Adverse effects will be analyzed using logistic regression. The time to resolve abdominal discomfort will be estimated using Kaplan-Meier analysis, and IUD insertion success will be compared using the chi-square test.

**Keywords:** contraceptive methods, intrauterine device (IUD), pain management, photobiomodulation therapy, randomized clinical trial

**CONTEXTUALIZAÇÃO**

A gestação não planejada acomete até 65% das mulheres (Costa *et al*., 2022) em algumas regiões do país, apesar das políticas públicas voltadas para a garantia do direito reprodutivo. A gravidez não planejada pode levar à realização de aborto em condições inseguras e à má assistência durante o pré-natal, que são causas importantes de mortalidade materna, por isso, fazem-se necessárias as ações de planejamento reprodutivo e garantia de acesso aos diversos métodos contraceptivos (Costa *et al*., 2022). Os métodos contraceptivos reversíveis de ação prolongada, como o dispositivo intrauterino (DIU) de cobre ajuda a reduzir o risco de gravidez indesejada em longo prazo, especialmente entre mulheres com menor escolaridade e baixo nível socioeconômico (Neto *et al*., 2021).

Segundo a Pesquisa Nacional de Saúde de 2019, entre as mulheres de 15 a 49 anos que ainda menstruavam e que tinham sido sexualmente ativas nos últimos 12 meses 40,6% usavam pílula anticoncepcional, 22,9% usavam algum método de esterilização (17,3% a laqueadura e 5,6% a vasectomia) e apenas 4,4% usavam DIU apesar de todos os seus aspectos positivos em relação aos outros métodos (Manual Técnico para Profissionais de Saúde,2018). Entre as indicações para o DIU, podemos citar o desejo de método anticoncepcional de longa duração para mulheres em idade reprodutiva, incluindo a adolescência. É um método anticoncepcional de emergência, podendo ser colocado a qualquer momento do ciclo; controla o sangramento uterino aumentado (DIU hormonal); controla a dismenorreia (DIU hormonal); é uma opção contraceptiva para mulheres com histórico pessoal e familiar de trombose; mantém a anticoncepção pré-captação de óvulos (reprodução assistida), sem piorar o desfecho do procedimento (Ministério da Saúde, 2018).

Podemos citar como contraindicações do DIU, a distorção importante da cavidade uterina, doença inflamatória pélvica ativa, gravidez conhecida ou suspeita, doença de Wilson ou alergia ao cobre e sangramento uterino anormal sem causa definida (Ministério da Saúde, 2018). Para inserção de um dispositivo intrauterino, é necessário que o profissional de saúde saiba que a mulher não está grávida e que ela não apresente nenhum sinal ou sintoma de gravidez. São utilizados alguns critérios para essa definição: A inserção pode ser feita durante a menstruação ou até 7 dias após o início da menstruação normal; não ter tido relações sexuais desde o início da última menstruação normal; ter usado corretamente e consistentemente um método confiável de contracepção; estar a menos de 7 dias após um abordo espontâneo ou induzido; dentro de 4 semanas no pós-parto; ou estar em aleitamento materno exclusivo ou quase exclusivo, em amenorreia e antes de 6 meses pós-parto (Ministério da Saúde, 2018).

**Inserção do DIU**

O DIU de cobre é um método contraceptivo que deveria ser amplamente ofertado e inserido nas Unidades Básicas de Saúde (UBS) e Ambulatórios Médicos de Especialidades (AME). No entanto, no Brasil, há escassez de estudos que documentem e analisem a implementação desse método nos serviços de saúde. Diante do receio da inserção e do uso do DIU (Coleman et al., 2024), tanto por parte das mulheres quanto dos profissionais de saúde, é fundamental promover pesquisas que aprofundem o conhecimento sobre os procedimentos de inserção deste dispositivo nos serviços de saúde. Estes estudos podem contribuir para desmistificar o método, garantindo maior adesão a essa forma de contracepção, que é eficaz e de baixo custo. (Almeida T, et al., 2023; Barreto D. et al,. 2020). Isto permitiria ampliar sua oferta, garantindo maior acesso à população de baixa renda (Almeida T, et al., 2023 ; Barreto D. et al,. 2020). Uma das grandes barreiras para a inserção de DIU, é a dor durante a inserção e consequente medo da paciente de passar por esse procedimento (Almeida et al., 2023; Lopes, et al., 2015).

A medula espinhal, é responsável pela inervação sensitiva de pele, músculos, articulações e vísceras e cada um desses grupos é chamado respectivamente de dermátomo, miótomo, esclerótomo ou viscerótomo. A dor no momento da inserção do DIU é de dois tipos: visceral e somática.

A dor visceral é produzida pela dilatação do colo do útero, pois os nervos aferentes estão localizados entre as fibras dos músculos do colo do útero e os impulsos nervosos são transmitidos para a coluna espinhal por nervos sensoriais que são acompanhados por nervos simpáticos, sendo os dermatomos de T10, T11, T12 e L1 (plexo hipogástrico superior) diretamente envolvidos na percepção da dor (Erdoğan *et al*., 2023).

A dor somática resulta da distensão da musculatura do assoalho pélvico, vagina e períneo, e os impulsos dolorosos são conduzidas pelos nervos pudendos, sendo os dermátomos S2, S3, S4, os mais importantes na percepção da dor (Erdoğan *et al*., 2023).

A neuroanatomia mostra que os nervos da vagina e o útero são derivados do plexo nervoso uterovaginal, que é um dos plexos pélvicos que se estendem do plexo hipogástrico inferior até as vísceras pélvicas. Fibras aferentes simpáticas, parassimpáticas e viscerais atravessam esse plexo. (Moore,2014)

A inervação simpática origina-se nos segmentos torácicos inferiores (T10) da medula espinal e atravessa os nervos esplâncnicos lombares e a série de plexos Inter mesentérico-hipogástrico-pélvicos. A inervação parassimpática origina-se nos segmentos S2–S4 da medula espinal e atravessa os nervos esplâncnicos pélvicos até o plexo hipogástrico inferior-uterovaginal. As fibras aferentes viscerais que conduzem impulsos de dor do fundo e do corpo do útero (acima da linha de dor pélvica) intraperitoneais seguem a inervação simpática retrógrada para chegar aos corpos celulares nos gânglios sensitivos de nervos espinais torácicos inferiores-lombares superiores. As fibras aferentes que conduzem impulsos de dor do colo do útero e da vagina (abaixo da linha de dor pélvica) subperitoneais seguem as fibras parassimpáticas retrogradamente através dos plexos uterovaginal e hipogástrico inferior e dos nervos esplâncnicos pélvicos para chegar aos corpos celulares nos gânglios sensitivos dos nervos espinais S2–S4. As duas diferentes vias seguidas por fibras de dor visceral são clinicamente importantes porque propiciam vários tipos de anestesia durante o parto, procedimentos pélvicos e controle de dores crônicas na região pélvica. Todas as fibras aferentes viscerais do útero e da vagina não relacionadas com a dor (aquelas que conduzem sensações inconscientes) também seguem a última via. (Moore, 2014)

Figura 2- Inervação da região uterina.

**Métodos analgésicos**

Já foram realizados diversos estudos para tentar controlar e diminuir o desconforto durante a inserção do DIU (Lopes *et al*., 2015; Neto ED da S et al, 2021; Almeida T. et al, 2023; Erdoğan P et al, 2023), mas sempre com resultados inconclusivos, seja com uso de anti-inflamatórios não hormonais (AINES) (Lopes *et al*., 2015; Neto ED da S et al, 2021; Almeida T. et al, 2023; Erdoğan P et al, 2023), uso de anestésicos locais (Lopes *et al*., 2015), ou preparo de colo (Lopes *et al*., 2015; Neto ED da S et al, 2021; Almeida T. et al, 2023; Erdoğan P et al, 2023).

Em revisão da Cohrane (Lopes *et al*., 2015), foi demonstrado que o gel de lidocaína a 2%, o misoprostol e a maioria dos AINEs (anti-inflamatórios não esteroides) não ajudaram a reduzir a dor e que essas intervenções são ineficazes, não necessitando novas pesquisas sobre o assunto. Algumas formulações de lidocaína, tramadol e naproxeno apresentaram algum efeito na redução da dor relacionada à inserção do DIU em alguns grupos específicos. A maior parte das evidências de eficácia foi de qualidade moderada, proveniente de ensaios isolados.

Diante da falta de evidências científicas sobre a eficácia da analgesia local para reduzir a dor na inserção de DIU, procuramos outras formas de analgesia para o controle da dor pélvica aguda e crônica, bem como no trabalho de parto. Foram encontrados trabalhos que mostram que o bloqueio paravertebral entre T10 e S4 com anestésicos, água destilada e fotobiomodulação apresentam algum nível de resultados. (Traverzim *et al*., 2018; Almeida *et al*., 2023; Neto *et al*., 2021). Alguns métodos de estimulação paravertebral têm sido eficazes no controle de dores pélvicas, especialmente durante o trabalho de parto. Entre eles, podemos citar a Estimulação Elétrica Nervosa Transcutânea (TENS): A TENS tem sido amplamente utilizada para analgesia no parto. Embora o mecanismo exato ainda não seja plenamente compreendido (Njogu et al., 2021), estudos comprovam sua eficácia e segurança. A aplicação da TENS de alta frequência (80 a 100 Hz) e largura de pulso de 350 microssegundos, com dois pares de eletrodos posicionados entre os níveis paravertebrais T10-L1 e S2-S4 durante a fase ativa do trabalho de parto, resultou em redução significativa da dor, conforme avaliado pela Escala Analógica Visual (VAS) (Soares et al., 2022).

Como método anestésico, também temos o bloqueio anestésico. O bloqueio anestésico paraespinhoso consiste em anestesiar o segmento espinhal entre T10 e L2, responsável pela inervação das vísceras pélvicas. Esse método proporciona alívio rápido para dores pélvicas agudas, mas não é indicado para o manejo da dor crônica (Rosa et al., 2013).

Em um estudo realizado em 2018 no Conjunto Hospitalar do Mandaqui, em colaboração com o serviço de Biofotônica da Uninove, a irradiação com LED vermelho e infravermelho na região paravertebral entre T10 e S4 demonstrou ser eficaz na analgesia durante o trabalho de parto, promovendo conforto e reduzindo a dor pélvica (Traverzim et al., 2018).

**Escolha do comparador**

A manipulação do colo e passagem do dispositivo pelo orifício interno podem ser desconfortáveis para algumas mulheres. O artigo de Tabatabaei et al. (2024) discute a dor associada à inserção de dispositivos intrauterinos (DIUs) como fator a ser considerado cuidadosamente para evitar complicações. Dores leves a moderadas são consideradas normais durante a inserção. Entretanto, dores severas ou persistentes são indicativas de complicações como perfuração uterina ou dano visceral. Profissionais de saúde não devem subestimar a dor relatada pelas pacientes ou não realizar os exames complementares necessários para verificar a correta posição do DIU, o que pode resultar em diagnósticos tardios e reinserções desnecessárias (Tabatabaei et al. 2024).

Anestesia mais profundas são contraindicadas, pois um pequeno nível de dor é desejável, como descrito acima. Algumas estratégias podem ser utilizadas, apesar de não terem eficácia científica comprovada, como a administração de antiinflamatório não esteróide (AINE) por via oral, antes da inserção do DIU. O grupo controle em trabalhos de inserção de DIU pode ser com placebo, ausência de intervenção ou outra intervenção ativa (Lopes, 2015).

**Fotobiomodulação no controle da dor**

Estudos recentes têm demonstrado a eficácia da fotobiomodulação no tratamento de dores lombares, utilizando diferentes comprimentos de onda. Lin et al. (2020) observaram bons resultados com a irradiação da região toracolombar, utilizando comprimentos de onda entre 630 e 850 nm. Em outro estudo, Tomazoni et al. (2020) empregaram 490 nm com correção para 570 nm e constataram alterações na microcirculação e no controle da liberação de interleucinas inflamatórias.

Adicionalmente, a aplicação de 905 nm durante 3 minutos, cobrindo uma área maior (T11 a S1), foi eficaz para o manejo da dor lombar crônica (Tomazoni et al., 2017). Em outro estudo, Traverzim (2018) utilizou irradiação entre T10 e S4 para analgesia no trabalho de parto, aplicando energia de 1,5 J em comprimentos de onda vermelho e infravermelho, com resultados promissores.

Dado que resultados promissores têm sido observados com o uso de terapias não medicamentosas para inserção de DIU (Gemzell-Danielsson, 2019) para analgesia durante o parto (Traverzim, 2018), e considerando que o Ministério da Saúde recomenda a inserção do DIU intrauterino sem o uso de qualquer recurso analgésico, torna-se essencial investigar formas de minimizar a dor relatada pelas mulheres durante e após a inserção do dispositivo. Estudos adicionais são necessários para explorar alternativas que possam reduzir esse desconforto, incentivando mais mulheres a aderirem a esse método contraceptivo, que é eficaz e de baixo custo, promovendo um impacto positivo na saúde pública brasileira.

**Justificativa**

A gravidez não planejada afeta até 65% das mulheres em algumas regiões do Brasil (Costa et al., 2022), apesar das políticas públicas voltadas para garantir o direito reprodutivo. Esse cenário aumenta o risco de abortos em condições não seguras e compromete a assistência pré-natal, constituindo causas significativas de mortalidade materna. Diante disso, é fundamental implementar ações de planejamento reprodutivo que assegurem o acesso a métodos contraceptivos eficazes (Ministério da Saúde 2018).

O dispositivo intrauterino (DIU) de cobre é um método contraceptivo reversível de longa duração, que se destaca por reduzir de forma expressiva o risco de gravidez indesejada, especialmente em mulheres com menor nível de escolaridade e baixa condição socioeconômica (Neto et al., 2021). Contudo, a dor durante e após a inserção do DIU é um fator que desencoraja muitas mulheres a optar por esse método, comprometendo a adesão e a continuidade do uso.

Os profissionais ainda precisam de intervenções melhores do que as geralmente utilizadas. Segundo uma revisão Cochrane de 2015 (Lopes, 2015) quase todos os ensaios utilizaram contraceptivos intrauterinos. A maior parte das evidências de eficácia foi considerada de qualidade moderada, pois se baseava em estudos isolados. O gel de lidocaína 2%, misoprostol e a maioria dos AINEs não reduziram a dor. Algumas formulações de lidocaína, tramadol e naproxeno apresentaram efeito na redução da dor relacionada à inserção do DIU em grupos específicos. Os autores concluem que intervenções sem efeito não precisam de novas investigações.

Neste contexto, torna-se necessário investigar alternativas para reduzir o desconforto associado à inserção do DIU, promovendo maior aceitação e adesão ao método. A fotobiomodulação (FBM), por sua comprovada ação anti-inflamatória e analgésica, surge como uma alternativa promissora. Ao irradiar as terminações nervosas da região toracolombar, a FBM pode inibir a propagação da dor para órgãos somáticos e viscerais, proporcionando um procedimento mais confortável e uma recuperação mais rápida.

Assim, este estudo justifica-se pela necessidade de avaliar se a utilização da FBM durante a inserção do DIU-Cu T 380 em sessão única pode contribuir para a redução da dor intra e pós-procedimento, promovendo maior conforto e bem-estar às mulheres e, consequentemente, encorajando a adoção de um método contraceptivo seguro e de baixo custo, com impacto positivo na saúde pública brasileira.

**Hipótese experimental**

O uso da FBM é capaz de diminuir a percepção da dor durante a inserção de DIU de cobre T 380 para contracepção.

## **OBJETIVOS**

### **2.1. Objetivo geral**

O objetivo deste estudo será avaliar se a fotobiomodulação aplicada de forma preventiva altera a percepção da dor durante a inserção de DIU de cobre T 380 para contracepção em mulheres sem comorbidades.

### **2.2. Objetivos específicos**

## Avaliação da dor (EVA) durante as fases de inserção do DIU (Pozzi, Histereometria e inserção do DIU) 5 e 15 minutos, 24 e 48 horas após a inserção do DIU.

## Quantidade de medicamentos (paracetamol) ingeridos pela paciente no período de 48 horas.

## Avaliação da ansiedade das pacientes por meio do instrumento Generalized Anxiety Disorder 7 (GAD-7)

## Avaliação da qualidade de vida das pacientes por meio do instrumento WHOQOL-Pain

## Satisfação das pacientes por meio de questionário estruturado (Lopes, 2015)

## Tempo para cessar o desconforto abdominal

## Efeitos adversos (ex: sangramento, desmaio, alergia)

## Efeitos colaterais (cólicas, calafrio, anestesia da língua)

## Falha na inserção do DIU

## **MATERIAL E MÉTODOS**

Trata-se de um ensaio clínico único-centro randomizado, duplo-cego controlado com dois grupos paralelos, de superioridade delineado conforme o os critérios SPIRIT *Statement* (<https://www.spirit-statement.org/>)

O projeto será submetido ao Comitê de Ética em Pesquisa (CEP) do Conjunto Hospitalar do Mandaqui da cidade de São Paulo. Qualquer intercorrência ou alteração durante o estudo será reportada e esclarecida ao CEP e nas futuras publicações deste estudo. Após explicação verbal pela pesquisadora principal, e por escrito do estudo, as participantes que aceitarem participar, assinarão o Termo de Consentimento Livre e Esclarecido (TCLE). As participantes que desejarem receber os dados da pesquisa, informarão o seu *e-mail* no TCLE e o artigo completo será fornecido assim que publicado. Os tratamentos serão realizados no Conjunto Hospitalar do Mandaqui, situado na Zona Norte da cidade de São Paulo, Brasil, no período de novembro 2024 a abril de 2026 pela médica Ginecologista com experiência de mais de 10 anos. O projeto será registrado na Plataforma *Clinicaltrials* (<https://clinicaltrials.gov/>).

**Calibração/treinamento**

Haverá apenas uma examinadora que avaliará 5 mulheres, que não farão parte do estudo. Serão realizadas avaliações clínicas de dor exatamente como proposto neste trabalho. A pesquisadora principal é médica ginecologista com experiência de mais de 10 anos na área e fará todas as inserções de DIU e as avaliações pós-operatórias. Neste trabalho, a pesquisadora principal será treinada para avaliar os seguintes desfechos: ansiedade, por meio do questionário Generalized Anxiety Disorder 7 (GAD-7); qualidade de vida, com o instrumento WHOQOL-Pain; e satisfação das pacientes, com base em um questionário estruturado, conforme o estudo de Lopes (2015).

**Cálculo do tamanho da amostra**

O tamanho total da amostra será de 72 pacientes por grupo. Esse valor foi calculado para fornecer um poder de 80% (α = 0,05) e um tamanho de efeito de 0,14. Para determinar o número de pacientes em cada grupo, foi realizado um cálculo amostral utilizando o software G*Power 3.1.9.7. A amostra foi calculada para 2 grupos. Serão realizadas 8 mensurações durante as fases de inserção do DIU (Pozzi, Histereometria e inserção do DIU) 5 e 15 minutos, 24 e 48 horas após a inserção do DIU.


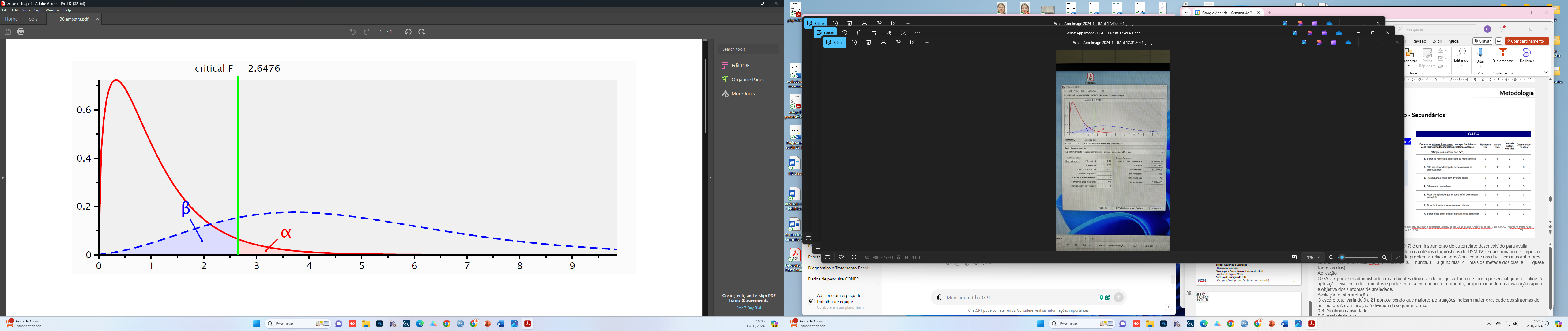


Fonte: software G*Power 3.1.9.7.

### **3.1. Descrição da amostra**

Serão selecionadas mulheres em idade reprodutiva, encaminhadas ao hospital para contracepção, das UBSs da Zona Norte de São Paulo ou do Pronto Socorro ginecológico do Conjunto Hospitalar do Mandaqui.

**Critérios de Inclusão/Exclusão**

Critérios de inclusão:

- Participantes de 18-50 anos,
- Sexo feminino,
- Sem predileção de raça ou nível socioeconômico,
- Nulíparas ou multíparas

Critérios de exclusão:

- Gravidez conhecida ou suspeita,
- Dor crônica diagnosticada,
- Infecção ativa local,
- Qualquer medicamento para dor nas últimas 12 horas,
- Contraindicação conhecida para a colocação do DIU (distorção importante da cavidade uterina, doença inflamatória pélvica ativa, doença de Wilson),
- Alergia ao cobre (Ministério da Saúde, 2018),
- Sangramento uterino anormal sem causa definida (Ministério da Saúde, 2018),
- Com qualquer alteração na região lombar como por exemplo: neoplasias ativas, osteomielite instalada, qualquer lesão tissular profunda preexistente, com necrose ou infectada,
- Com histórico de fotossensibilidade.
- Pacientes com IMC ≥25 kg/m^2^ (OMS, 2000)

**Critérios de interrupção do estudo**

Não há planos para interromper ou modificar as intervenções alocadas para uma determinada participante do ensaio pois o grupo controle será realizado o tratamento preconizado pelo Ministério da Saúde, por uma revisão sistemática Cochrane (Lopes et al., 2015) e recomendada pelo fabricante do dispositivo intra-uterino (DIU). Até o momento não há evidencia de tratamento para analgesia na inserção do dispositivo intra-uterino (Lopes, et al.,2015). No grupo Experimental será realizado um procedimento que não causa nenhum efeito adverso ou colateral, há relato de analgesia durante o trabalho de parto (Traverzim et al., 2018). Portanto, como o estudo não oferece nenhum risco para as pacientes, não há critério para interrupção do estudo.

**Recrutamento**

O recrutamento será de livre demanda, com participantes encaminhadas ao hospital para contracepção, das UBSs da Zona Norte de São Paulo ou do Pronto-Socorro ginecológico do Conjunto Hospitalar do Mandaqui.

**Aplicação de Anamnese e Coleta de Fatores de Risco**

Será realizada a anamnese (ANEXO 1) em todas as pacientes. Além das perguntas relacionadas à saúde geral, serão coletados dados demográficos (idade, gênero, estado civil, ocupação, nível educacional, salário), dados da história médica (queixa principal, estado da doença atual, história médica, medicamentos em uso) presença de tabagismo ou etilismo, uso de antibioticoterapia sistêmica nos últimos 12 (doze) meses, hospitalização ou infecção (incluindo de pele) nos últimos 12 (doze) meses se já utilizou DIU anteriormente, métodos anticoncepcionais que costuma utilizar.

Esse questionário nos permitirá correlacionar fatores de riscos clínicos, demográficos e epidemiológicos que possam relacionar-se aos resultados obtidos nesta população.

**Alocação (geração da sequencia, mecanismo de ocultação de alocação, implemnentação)**

Será randomizado o tratamento realizado imediatamente após a realização da colocação do DIU, que poderá ser a fotobiomodulação ou a simulação da fotobiomodulação. Será usado um programa disponível na internet e gerador de sequência aleatória (<https://www.sealedenvelope.com/>) e será selecionada a opção de randomização por blocos de 2 tratamentos na proporção de 1:1, ou seja, com a mesma quantidade de pacientes nos dois grupos (n=36). Como o estudo possui uma amostra total de 72 pacientes serão realizados 12 blocos de 6 pacientes. Envelopes opacos serão identificados com números sequenciais (de 1 a 72) e no seu interior será inserida a informação do grupo experimental correspondente conforme a sequência aleatória gerada. Os envelopes serão selados e permanecerão lacrados em um lugar seguro até o momento da inserção do DIU. Apenas esse profissional saberá a natureza dos tratamentos. O envelope será novamente selado e permanecerá junto com o prontuário da paciente e somente será revelado ao final da análise estatística. A geração da sequência aleatória e a preparação dos envelopes serão realizadas por uma pessoa não envolvida diretamente ao estudo.

Todas serão submetidas ao mesmo protocolo de inserção com o mesmo profissional como descrito no item “Protocolo de inserção do DIU”. Imediatamente após o término da colocação, o pesquisador responsável pela aplicação da FBM retirará e abrirá 1 envelope (sem alterar a sequência numérica dos demais envelopes) e realizará o procedimento indicado ou a sua simulação. Apenas esse pesquisador saberá a natureza dos tratamentos.

O pesquisador responsável pela pesquisa ficará responsável pelo armazenamento (plataformas digitais) e tabulação das informações O estaticista será responsável pela análise dos dados obtidos ao fim da pesquisa e será cego quanto às intervenções. Esses mesmos envelopes conterão as fichas das participantes (questionário de anamnese, dados demográficos e fatores de risco, e o TCLE) e serão guardadas em arquivo.

**Cegamento do Estudo (mascaramento)**

Apenas o pesquisador responsável pela realização dos tratamentos (que abrirá os envelopes da randomização) saberá qual tratamento atribuído a cada paciente. A paciente será cega em vista à similaridade dos tratamentos (LED ligado/LED desligado). O pesquisador responsável pela coleta dos desfechos será cego para as intervenções realizadas pois não estará na sala no momento da aplicação do tratamento. A identificação de cada grupo será revelada apenas após análise estatística dos dados para todos os envolvidos no estudo por este pesquisador. Portanto, o pesquisador responsável pela coleta de dados, e o estaticista também serão cegos quanto aos tratamentos atribuídos aos grupos. Durante o ensaio o desmascaramento do estudo não está previsto por não haver nenhum tipo de intercorrência prevista para o uso da FBM.

**Protocolo de inserção do DIU segundo as recomendações do Ministério da Saúde**

Antes do procedimento, as etapas serão explicadas novamente para a paciente para reduzir a ansiedade e promover maior relaxamento durante a inserção do DIU. Será realizado um exame pélvico bimanual para avaliar o tamanho, posição e mobilidade do útero. Medidas de prevenção de infecção serão adotadas, utilizando luvas estéreis e realizando a limpeza do colo uterino com antisséptico à base de cloridrato de clorexidina. Em seguida, será feito o pinçamento delicado do lábio anterior do colo uterino com a pinça Pozzi para estabilização durante a inserção. O histerômetro será introduzido de forma lenta e cuidadosa para determinar a profundidade e a angulação uterina, minimizando o risco de perfuração. Durante a preparação do DIU, os instrumentos e luvas serão mantidos estéreis, e o dispositivo será montado conforme as orientações do fabricante, mantendo as hastes na posição horizontal durante a inserção. O DIU será inserido utilizando a técnica retrátil: o tubo de inserção será introduzido até o fundo uterino e, em seguida, retirado parcialmente, mantendo o êmbolo fixo para liberar as hastes do DIU. Após alguns segundos, o êmbolo e o tubo-guia serão removidos. Os fios do DIU serão cortados, deixando-os com 2 a 3 cm de comprimento em relação ao colo uterino, e a paciente permanecerá deitada por cerca de 15 minutos após o procedimento para reduzir o desconforto. Após a inserção, a paciente será monitorada para avaliar seu bem-estar e possíveis reações vasovagais (como suor, vômito ou desmaios), que são raras e autolimitadas. Será registrado no prontuário o comprimento dos fios do DIU em relação ao colo uterino, e a paciente será orientada a retornar para consulta de seguimento em 30 dias para verificar a posição do dispositivo e avaliar a adaptação.

### **Composição dos grupos**

As 72 participantes serão alocadas nos grupos Controle e experimental da seguinte forma:

**Grupo Controle –Simulação da FBM (n=36 participantes):** Todas as participantes serão submetidas ao mesmo procedimento de colocação do DIU convencional conforme descrito anteriormente. As participantes receberão a simulação da FBM e serão atendidas de forma idêntica ao grupo Experimental. O pesquisador responsável pela aplicação da FBM irá simular as irradiações posicionando o dispositivo no mesmo local descrito para o grupo FBM, porém, o equipamento será mantido desligado. Para que a participante não identifique o grupo ao qual pertence, o som de ativação do equipamento (bip) será gravado e ligado na hora da aplicação.

**Grupo Experiemtal – FBM (n=36 participantes):** Todas as participantes serão submetidas ao mesmo procedimento. Para a irradiação será utilizada uma placa de LEDs da marca Sportlux® (Brasil, SP) (Figura 1) com as seguintes especificações. A descrição do equipamento, parâmetros dosimétricos e número de aplicações de FBM estão descritos no Quadro 1.

**Tabela 1:** Parâmetros dosimétricos utilizados para a FBM preventiva.

| **PARÂMETROS TÉCNICOS** | **Sportllux®** |
| --- | --- |
| Fonte de luz | LED |
| Técnica de aplicação | Contato |
| Comprimento de onda | 132 LEDs com 660nm |
|  | 132 LEDs com 850nm |
| Banda espectral | 20 nm |
| Área do feixe no alvo | 0,5 cm² |
| Tempo de irradiação | 20min |
| Potência média de cada LED | 8mW |
| Irradiância | 16 mW/cm² |
| Tempo de aplicação | 10 minutos |
| Energia por LED | 4,8J |
| Exposição radiante | 9,6J/cm² |
| Ângulo de emissão da luz | 120° |

nm- nanometro, W- Watt, J- Joule,


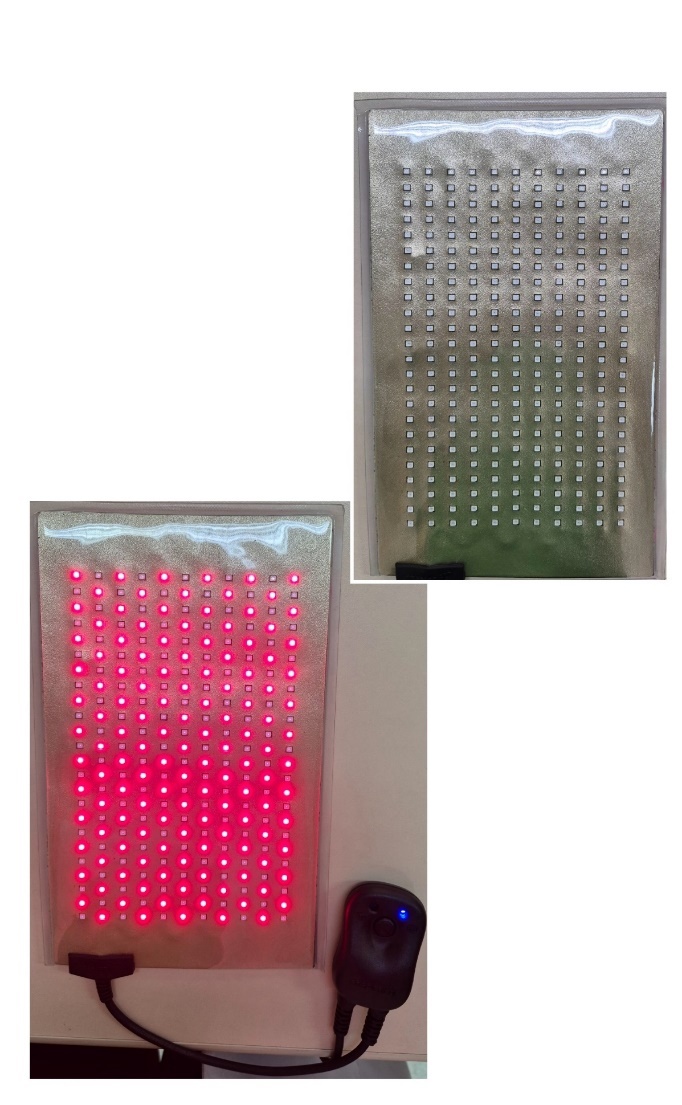

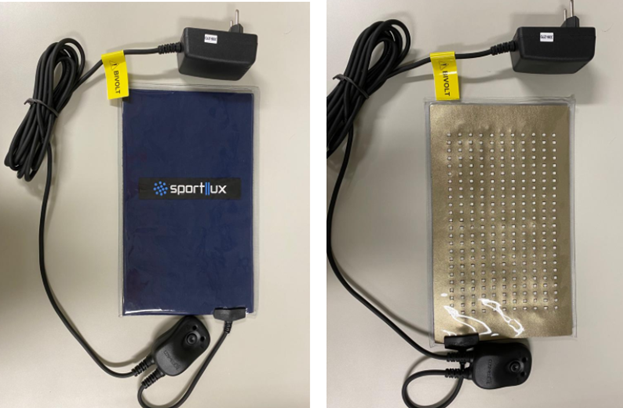

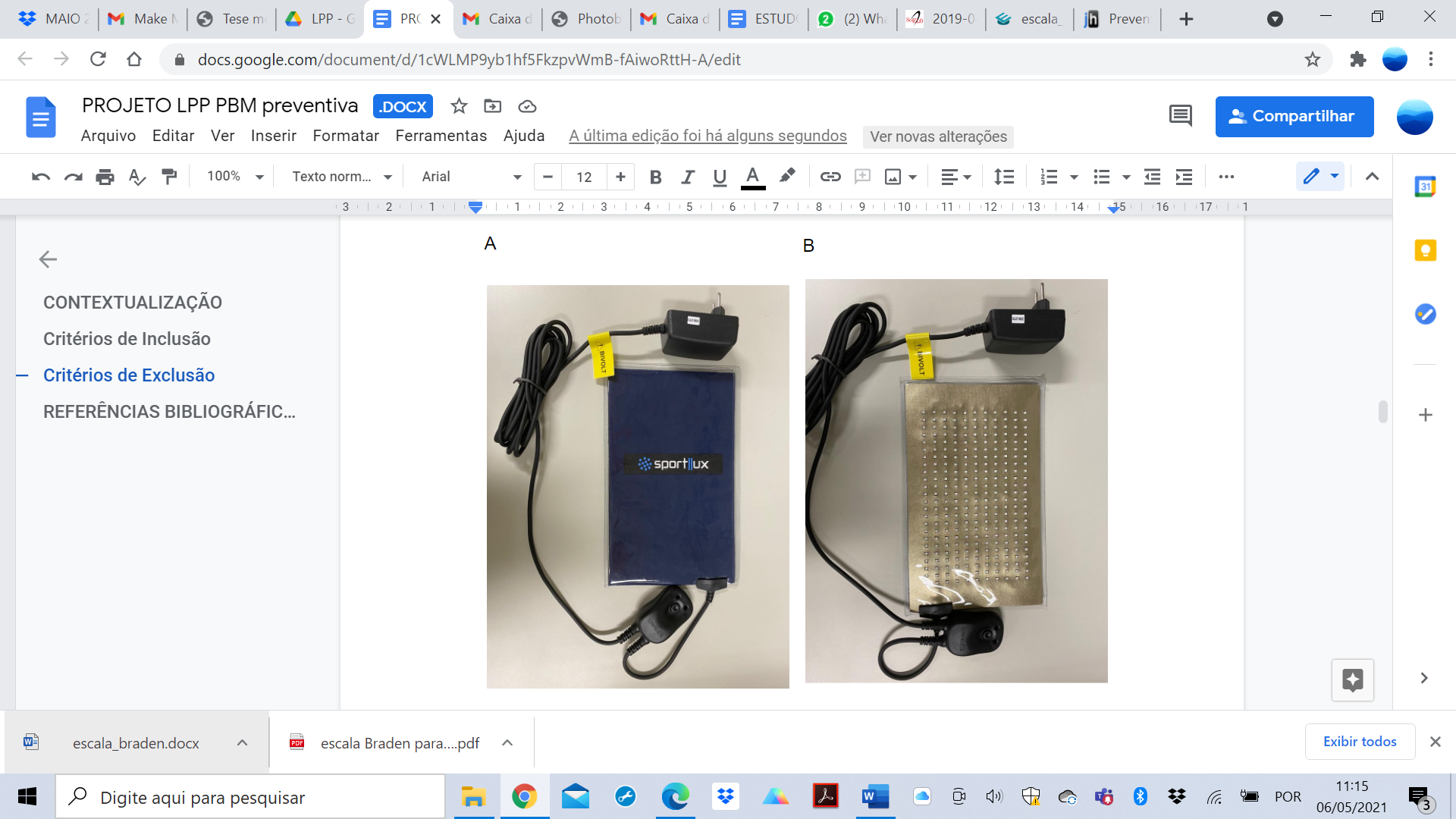


C

B

A

Figura 1: Placa de LEDs da marca Sportlux® utilizada para irradiação preventiva na inserção do DIU. Figura A e B. Placa desligada. Figura C: Placa ligada.

A região irradiada abrangerá a coluna lombar e toráxica, mais especificamente de T10 a L4. utilizando a placa de LED no sentido vertical.

Figura 2. Fluxograma do estudo

Recrutamento

Treinamento dos pesquisadores

Seleção dos participantes conforme critérios de inclusão

Convite para participar e assinatura do termo de consentimento livre e esclarecido (TCLE)

(Estimativa de 10% de evasão do estudo)

Aleatorização

*Baseline*  (T0)

EVA, entrega da cartela de analgésicos aplicação dos questionários de satisfação e qualidade de vida e ansiedade

*Baseline*  (T0)

EVA, entrega da cartela de analgésicos aplicação dos questionários de satisfação e qualidade de vida e ansiedade

Inserção do DIU CU T 380 + FBM

Inserção do DIU CU T 380 + FBM placebo

(n=xxx)

1^o^ avaliação (T1=6h)

EVA, contagem de analgésicos aplicação dos questionários de satisfação e qualidade de vida e ansiedade

1^o^ avaliação (T1=6h)

EVA, contagem de analgésicos aplicação dos questionários de satisfação e qualidade de vida e ansiedade

2^o^ dia de avaliação (T2=24h)

EVA, contagem de analgésicos aplicação dos questionários de satisfação e qualidade de vida e ansiedade

2^o^ dia de avaliação (T2=24h)

EVA, contagem de analgésicos aplicação dos questionários de satisfação e qualidade de vida e ansiedade

3^o^ dia de avaliação (T3=48h)

EVA, contagem de analgésicos aplicação dos questionários de satisfação e qualidade de vida e ansiedade

3^o^ dia de avaliação (T3=48h)

EVA, contagem de analgésicos aplicação dos questionários de satisfação e qualidade de vida e ansiedade

3^o^ dia de avaliação (T3=48h)

EVA, contagem de analgésicos aplicação dos questionários de satisfação e qualidade de vida e ansiedade

3^o^ dia de avaliação (T3=48h)

EVA, contagem de analgésicos aplicação dos questionários de satisfação e qualidade de vida e ansiedade

**Variáveis de desfecho do estudo**

- Dor – Avaliação por meio da Escala Visual Analógica (EVA) no baseline, durante as fases de inserção do DIU (Pozzi, Histereometria e inserção do DIU) 5 e 15 minutos, 24 e 48 horas após a inserção do DIU
- Descrição da avaliação da dor – Livre relato
- Quantidade de analgésicos ingeridos - (paracetamol) ingeridos pela paciente após a inserção de DIU de cobre T 380 no período que vai do baseline até as 48 horas após a inserção.
- Avaliação da ansiedade das pacientes submetidas à inserção de DIU de cobre T 380 para contracepção por meio do instrumento *Generalized Anxiety Disorder 7*  (GAD-7) (Bártolo A, 2017) no período logo após a inserção (15 minutos).
- Avaliação da qualidade de vida por meio do instrumento WHOQOL-Pain das pacientes submetidas à inserção de DIU de cobre T 380 para contracepção **(Pedroso et al 2015)** no período que vai do baseline até as 48 horas após a inserção.
- Satisfação das pacientes submetidas à inserção de DIU de cobre T 380 para contracepção por meio de questões estruturadas (Lopes, et al 2015) no período logo após a inserção (15 minutos)
- Tempo necessário para cessar o desconforto abdominal (cólica menstrual) medido em dias no período que vai do baseline até as 48 horas após a inserção.
- Ultrassom para verificar o sucesso de colocação. Se houver falha na inserção do DIU. Será avaliado de forma dicotômica (Sucesso/ insucesso) no período que vai do baseline até as 48 horas após a inserção.
- Efeitos adversos (Perfuração Uterina, Deslocamento do DIU, Dor Abdominal e Sangramento Vaginal aumentado, alergia). Será realizada uma pergunta aberta para que a paciente responda abertamente sobre os efeitos adversos e depois serão elencados nominalmente para que ela possa se lembrar de algum efeito que porventura tenha esquecido de relatar.
- Efeitos colaterais (cólicas, dor leve, sangramento leve, anestesia da língua). Será realizada uma pergunta aberta para que a paciente responda abertamente sobre os efeitos colaterais e depois serão elencados nominalmente para que ela possa se lembrar de algum efeito que porventura tenha esquecido de relatar.

**Dor - Escala Visual Analógica (EVA)**

Será avaliada por meio da aplicação da Escala Visual Analógica (EVA) conforme o desenho abaixo. Um dos extremos apresenta a indicação '0', e o outro '10' que significa respectivamente 'sem dor' e 'dor insuportável'. Essa régua será a mesma para todas as participantes. As instruções sobre a marcação serão dadas sempre pelo mesmo operador. O valor da dor será contabilizado por meio de scores. Essa análise ocorrerá em todas as consultas (durante as fases de inserção do DIU -Pozzi, Histereometria e inserção do DIU- 5 e 15 minutos, 24 e 48 horas após a inserção do DIU.). As participantes serão questionadas sobre a dor espontânea em três momentos distinos durante a inserção: Dor no Pozzi (pinçamento) e dor na histerometria (medida do comprimento do Útero) e dor na inserção propriamente dita. Todas as pacientes serão lembradas por telefonemas diários para registrar seus desfechos conforme recomendado (Qamrudin 2014).

### *Pinçamento do Colo:* Realizar o pinçamento delicado do lábio anterior do colo uterino com a pinça Pozzi para estabilização durante a inserção.

### *Histerometria:* Introduzir o histerômetro de forma lenta e cuidadosa para determinar a profundidade e a angulação uterina, minimizando o risco de perfuração uterina.

- *Inserção na cavidade uterina:*

**Descrição da avaliação da dor – Livre relato**

Logo após a inserção do DIU será pedido às pacientes que relatem sua experiencia com a colocação e seu relato será anotado com as próprias palavras da participante para uma avaliação qualitativa.

**Quantidade de analgésicos ingeridos - (paracetamol) ingeridos pela paciente após a inserção de DIU de cobre T 380 no baseline, durante a inserção, 5 min, 15 min 24 h 48 h após a inserção.**

A quantidade de analgésicos ingeridos será avaliada conforme proposto por Bauer 2013. No início da pesquisa, será entregue para cada participante uma cartela de paracetamol® (fármaco com efeito puramente analgésico) (Jóźwiak-Bebenista, 2014), devendo ser guardada até o final do estudo e seu uso será liberado apenas em caso de dor. Ao fim do experimento, a quantidade de comprimidos será avaliada como outro parâmetro de mensuração da dor. O analgésico utilizado será paracetamol 500mg. Deverá ser utilizado apenas se houver dor. A dose recomendada caso haja necessidade será de 1 comprimido a cada 6 horas. A participante será solicitada a registrar por escrito a quantidade de analgésicos ingeridos, o dia e o horário. Será realizado um procedimento para monitorar a aderência dos participantes (será pedido as participantes levarem a cartela de analgésicos para a consulta para conferência). O uso de medicamentos será monitorado no período que vai do baseline até as 48 horas após a inserção.

**Avaliação da ansiedade das pacientes submetidas à inserção de DIU de cobre T 380 para contracepção por meio do instrumento *Generalized Anxiety Disorder 7-item* (GAD-7) no baseline e 5 min após o término do procedimento (Bártolo A, 2017).**

O *Generalized Anxiety Disorder 7* (GAD-7) é um instrumento de autorrelato desenvolvido para avaliar sintomas de ansiedade generalizada, baseado nos critérios diagnósticos do DSM-IV. O questionário é composto por sete itens, que mensuram a frequência de problemas relacionados à ansiedade nas duas semanas anteriores, com base em uma escala Likert de 4 pontos (0 = nunca, 1 = alguns dias, 2 = mais da metade dos dias, e 3 = quase todos os dias). O GAD-7 pode ser administrado em ambientes clínicos e de pesquisa, tanto de forma presencial quanto online. A aplicação leva cerca de 5 minutos e pode ser feita em um único momento, proporcionando uma avaliação rápida e objetiva dos sintomas de ansiedade.

O escore total varia de 0 a 21 pontos, sendo que maiores pontuações indicam maior gravidade dos sintomas de ansiedade. A classificação é dividida da seguinte forma:

- 0-4: Nenhuma ansiedade
- 5-9: Ansiedade leve
- 10-14: Ansiedade moderada
- 15-21: Ansiedade grave

O GAD-7 apresenta boa validade e confiabilidade, com uso extensivo em populações clínicas e não-clínicas. A estrutura do questionário pode ser analisada por meio de um modelo unifatorial ou bifatorial (itens somáticos e cognitivo-emocionais), dependendo do contexto de aplicação.

**Avaliação da qualidade de vida das pacientes submetidas à inserção de DIU de cobre T 380 para contracepção por meio do instrumento** WHOQOL-Pain **baseline e 48h após o término do procedimento.**

O WHOQOL-Pain é um módulo adicional ao instrumento WHOQOL-100, desenvolvido especificamente para avaliar a qualidade de vida em pessoas que convivem com dor crônica. Após um estudo preliminar, foram identificadas dez novas facetas relacionadas à dor e ao desconforto. Destas, quatro facetas foram selecionadas para compor a versão final do instrumento: **Alívio da dor, Raiva e frustração, Vulnerabilidade, medo e preocupação e Incerteza (Pedroso et al 2015).** Cada faceta é composta por quatro questões que abordam diferentes dimensões da experiência com dor. As respostas seguem uma escala do tipo Likert com cinco alternativas, que medem aspectos como intensidade, capacidade, avaliação e frequência. As pontuações são transformadas para uma escala de 0 a 100, permitindo uma interpretação quantitativa dos níveis de dor e seu impacto na qualidade de vida. s questões do WHOQOL-Pain não são integradas ao WHOQOL-100, sendo aplicadas separadamente ao final do questionário principal. Essa estrutura facilita a análise individual das facetas relacionadas à dor, permitindo uma avaliação específica e detalhada das experiências de dor crônica física.

**Satisfação das pacientes submetidas à inserção de DIU de cobre T 380 para contracepção por meio de perguntas norteadoras (Lopes, et al 2015) após o término do procedimento (15 minutos).**

- A experiencia de inserção foi desprazeirosa?
- Você faria novamente no futuro?
- Recomenda para alguma amiga?

**Período de tempo para cessar o desconforto abdominal após FBM medido em horas.**

Será anotado o tempo que a cólica se inicia até o momento que ela termina e não há mais nenhuma dor presente. Esse desfecho será medido em horas.

**Ultrassom para verificar o sucesso de colocação**

Se houver falha na inserção do DIU. Será avaliado de forma dicotômica (Sucesso/ insucesso).

**Efeitos adversos** **(Perfuração Uterina, Deslocamento do DIU, Dor Abdominal e Sangramento Vaginal aumentado, alergia).**

Será realizada uma pergunta aberta para que a paciente responda abertamente sobre os efeitos adversos e depois serão elencados nominalmente para que ela possa se lembrar de algum efeito que porventura tenha esquecido de relatar.

**Efeitos colaterais (cólicas, dor leve, sangramento leve, anestesia da língua).**

Será realizada uma pergunta aberta para que a paciente responda abertamente sobre os efeitos colaterais e depois serão elencados nominalmente para que ela possa se lembrar de algum efeito que porventura tenha esquecido de relatar.

**Aspectos éticos**

O presente estudo garante conformidade com as diretrizes éticas e regulatórias aplicáveis. Não estão previstas modificações no protocolo de pesquisa, mas caso haja alguma, será comunicada prontamente às partes interessadas, incluindo os investigadores, comitê de ética (envio de emenda), participantes, registros de ensaios clínicos

O consentimento livre e esclarecido será obtido por um pesquisador treinado, que fornecerá aos participantes informações detalhadas sobre os objetivos, procedimentos, riscos e benefícios do estudo, utilizando um termo de consentimento redigido em linguagem clara e acessível. Não haverá coleta de materiais biológicos nem mesmo armazenamento.

A coleta, o compartilhamento e o armazenamento das informações pessoais dos participantes seguirão protocolos rigorosos de proteção de dados para garantir a confidencialidade antes, durante e após o ensaio. Todos os dados serão anonimizados e armazenados de forma segura para prevenir acessos não autorizados. Não há conflitos de interesse financeiros ou de qualquer outra natureza. Apenas a pesquisadora principal terá acesso ao conjunto final de dados do estudo.

O pesquisador responsável se compromete a divulgar os resultados do estudo aos participantes, profissionais de saúde e ao público em geral, por meio de publicações, apresentações de trabalhos em congressos e respeitando quaisquer restrições de publicação previamente acordadas.

**Análise estatística**

A análise estatística será realizada utilizando o software *Statistical Package for the Social Sciences* (SPSS) versão 24.0 ou software equivalente. O nível de significância será estabelecido em 5% (p < 0,05) para todos os testes. A normalidade dos dados será avaliada pelo teste de Shapiro-Wilk. Variáveis contínuas serão descritas por meio de média e desvio-padrão, ou mediana e intervalo interquartílico, conforme a distribuição dos dados. Variáveis categóricas serão apresentadas como frequências absolutas e relativas.

Para a comparação entre os grupos experimental (FBM ativa) e controle (simulação de FBM) será utilizado o teste de Mann-Whitney para variáveis contínuas com distribuição não normal. Variáveis categóricas serão comparadas utilizando o teste qui-quadrado ou teste exato de Fisher, conforme valor mínimo esperado seja menor que 5.

A análise das variáveis relacionadas à dor (intensidade medida pela Escala Visual Analógica – EVA) em diferentes momentos (baseline, durante a inserção, 5 e 15 minutos, 24 e 48 horas após o procedimento) será realizada por meio do teste de Friedman ou por modelo linear generalizado, ajustando-se para possíveis covariáveis.

Para avaliar a ansiedade (GAD-7), qualidade de vida (WHOQOL-100) e satisfação com o procedimento, será realizado o teste Mann-Whitney para comparação entre os grupos nos diferentes momentos de avaliação.

O uso de analgésicos pós-procedimento será comparado entre os grupos por meio de análise de variância (ANOVA) para medidas repetidas, considerando o número de comprimidos ingeridos em cada momento (24h e 48h). Adicionalmente, para verificar a associação entre os grupos e a ocorrência de efeitos adversos e colaterais, será realizada regressão logística, considerando o grupo como variável preditora e os efeitos adversos como variável resposta.

Por fim, será realizada uma análise de Kaplan-Meier para estimar o tempo necessário para cessar o desconforto abdominal (cólica) e o sucesso de inserção do DIU será analisado por meio de comparação de proporções entre os grupos utilizando teste qui-quadrado.

**Referências bibliográficas**

Almeida, Tiago & Mata, André & Leitão, Cláudia & Basto, Daniela & Ferreira, Mariana. (2023). Management of pain related to the intrauterine devices insertion: what is the evidence?. 17. 287-298.

Barreto D da S, Maia DS, Gonçalves RD, Soares R de S. Dispositivo Intrauterino na Atenção Primária a Saúde: uma revisão integrativa. Rev Bras Med Fam Comunidade [Internet]. 30º de maio de 2021 [citado 15º de julho de 2024];16(43):2821. Disponível em: <https://rbmfc.org.br/rbmfc/article/view/2821>

Bártolo A, Monteiro S, Pereira A. Factor structure and construct validity of the Generalized Anxiety Disorder 7-item (GAD-7) among Portuguese college students. Cad Saude Publica. 2017 Sep 28;33(9):e00212716. doi: 10.1590/0102-311X00212716. PMID: 28977285.

Brasil. Ministério da Saúde. Secretaria de Atenção à Saúde. Departamento de Ações Programáticas Estratégicas. [Manual Técnico para Profissionais de Saúde : DIU com Cobre TCu 380A](https://portaldeboaspraticas.iff.fiocruz.br/wp-content/uploads/2018/12/manual_diu_08_2018.pdf) / Ministério da Saúde, Secretaria de Atenção à Saúde. Departamento de Ações Programáticas Estratégicas. – Brasília : Ministério da Saúde, 2018.

Coleman K, Carter C. Pain control during intrauterine device insertion: Transcutaneous electrical nerve stimulation. J Am Assoc Nurse Pract. 2024 Oct 1;36(10):594-596. doi: 10.1097/JXX.0000000000001031. PMID: 38787344.

Costa, Ana & Lanhoso, António & Capela, Eunice & Neves, Joaquim & Reis, José & Silva, Daniel. (2022). Pain management in office gynecological procedures. 16. 264-272.

Erdoğan P, Yardımcı H. Analgesic effects of LI4 acupuncture during intrauterine device insertion: a randomized controlled clinical trial. Arch Gynecol Obstet. 2023 Oct;308(4):1361-1368. doi: 10.1007/s00404-023-07106-5. Epub 2023 Jul 19. PMID: 37466690.

Durnin JV, Womersley J. Body fat assessed from total body density and its estimation from skinfold thickness: measurements on 481 men and women aged from 16 to 72 years. Br J Nutr. 1974 Jul;32(1):77-97. doi: 10.1079/bjn19740060. PMID: 4843734.

Gemzell-Danielsson K, Jensen JT, Monteiro I, Peers T, Rodriguez M, Di Spiezio Sardo A, Bahamondes L. Interventions for the prevention of pain associated with the placement of intrauterine contraceptives: An updated review. Acta Obstet Gynecol Scand. 2019 Dec;98(12):1500-1513. doi: 10.1111/aogs.13662. Epub 2019 Jun 27. PMID: 31112295; PMCID: PMC6900125.

Lin YP, Su YH, Chin SF, Chou YC, Chia WT. Light-emitting diode photobiomodulation therapy for non-specific low back pain in working nurses: A single-center, double-blind, prospective, randomized controlled trial. Medicine. 7 de agosto de 2020;99(32):e21611.

Lopez LM, Bernholc A, Zeng Y, Allen RH, Bartz D, O'Brien PA, Hubacher D. Interventions for pain with intrauterine device insertion. Cochrane Database Syst Rev. 2015 Jul 29;2015(7):CD007373. doi: 10.1002/14651858.CD007373.pub3. PMID: 26222246; PMCID: PMC9580985.

Manual Técnico para Profissionais de Saúde : DIU com Cobre TCu 380A / Ministério da Saúde, Secretaria de Atenção à Saúde. Departamento de Ações Programáticas Estratégicas. – Brasília : Ministério da Saúde, 2018.

Mc Mahon SB Koltzenburg M (eds). Wall and Melzack’s Textbook of pain 5 ed Elsevier, Londres, Reino Unido, 2006

Moore AFD. Anatomia orientada para a clínica. Editora Guanabara Koogan Ltda; 2014, Jan 2019.

Neto ED da S, Júnior ASM, Silva THCM, Ribeiro CBL., Nogueira J, Moreira L. Evaluation of oral ketorolac trometamol for pain control during intrauterine device insertion. Brazilian Journal of Development. 2001, 7(10), 95663–95672. <https://doi.org/10.34117/bjdv7n10-064a>

Njogu A, Qin S, Chen Y, Hu L, Luo Y. The effects of transcutaneous electrical nerve stimulation during the first stage of labor: a randomized controlled trial. BMC Pregnancy Childbirth. 2021 Feb 24;21(1):164. doi: 10.1186/s12884-021-03625-8. PMID: 33627077; PMCID: PMC7905652.

World Health Organization (WHO). (2000). Obesity: Preventing and Managing the Global Epidemic. Report of a WHO Consultation (WHO Technical Report Series 894). Geneva: World Health Organization.

Pedroso, Bruno & Gutierrez, Gustavo & Picinin, Claudia. (2016). WHOQOL-Pain: um instrumento de avaliação da qualidade de vida para pessoas que convivem com dor crônica física. Revista Brasileira de Qualidade de Vida. 8. 10.3895/rbqv.v8n3.4522.

Sikandar S, Dickenson AH. Visceral pain: the ins and outs, the ups and downs. Curr Opin Support Palliat Care. 2012 Mar;6(1):17-26. doi: 10.1097/SPC.0b013e32834f6ec9. PMID: 22246042; PMCID: PMC3272481.

Soares AM. Tópicos Especiais em Ciências da Saúde: teoria, métodos e práticas 6 [Internet]. 1^o^ ed. AYA Editora; 2022 [citado 31 de maio de 2024]. Disponível em: <https://ayaeditora.com.br/Livro/20605>

Tomazoni SS, Costa L da CM, Guimarães L de S, Araujo AC, Nascimento DP, Medeiros FC de, et al. Effects of photobiomodulation therapy in patients with chronic non-specific low back pain: protocol for a randomised placebo-controlled trial. BMJ Open. 1^o^ de outubro de 2017;7(10):e017202.

Traverzim MADS, Makabe S, Silva DFT, Pavani C, Bussadori SK, Fernandes KSP, et al. Effect of led photobiomodulation on analgesia during labor: Study protocol for a randomized clinical trial. Medicine. junho de 2018;97(25):e11120.
