# Supplementary material for: Photobiomodulation for pain management during placement of the copper T 380 intrauterine device: Protocol for a randomized, double-blind controlled trial
Source: PLoS One. 2026 May 28;21(5):e0349031. doi: 10.1371/journal.pone.0349031 (PMC13218537; doi:10.1371/journal.pone.0349031)
Supplement: S3 File — This is the S5 Fig legend; there is no legend. (DOCX) [file pone.0349031.s003.docx]

**NINTH OF JULY UNIVERSITY**

**Postgraduate Program in Biophotonics Applied to Health Sciences**

**Anna Carolina** **Nunes Ferraz**

**EFFECT OF PHOTOBIOMODULATION ON REDUCING PAIN PERCEPTION DURING INSERTION OF COPPER IUD T 380 FOR CONTRACEPTION:** **RANDOMIZED CONTROLLED CLINICAL TRIAL**

**São Paulo**

**2024**

**NINTH OF JULY UNIVERSITY**

**Postgraduate Program in Biophotonics Applied to Health Sciences**

**Anna Carolina Nunes Ferraz**

**EFFECT OF PHOTOBIOMODULATION ON REDUCING PAIN PERCEPTION DURING INSERTION OF COPPER IUD T 380 FOR CONTRACEPTION:** **RANDOMIZED CONTROLLED CLINICAL TRIAL**

|  | Research project submitted to the Research Ethics Committee of the Mandaqui Hospital Complex.  Advisor: Professor Dr. Anna Carolina Ratto Tempestini Horliana.  Co-Supervisor: Kristianne Porta Santos Fernandes |
| --- | --- |

**São Paulo**

**2024**

**Summary**

Unplanned pregnancy affects up to 65% of women in some regions of Brazil, increasing the risk of unsafe abortions and contributing to maternal mortality. The copper IUD is an effective and long-lasting contraceptive alternative, but its use is still limited in Brazil, reaching only 4.4% of women of reproductive age. One of the main barriers is the pain associated with its insertion, which generates fear and low adherence to the method. As the pain can be of visceral or somatic origin, traditional approaches such as anti-inflammatories and anesthetics show inconclusive results in reducing this discomfort. Photobiomodulation (PBM) promotes anti-inflammatory and analgesic effects, showing positive results in controlling pelvic pain in other clinical contexts, such as labor. The aim of this study is to evaluate the effectiveness of biofeedback breathing (BFB) as a preemptive analgesic method for insertion of the T380 copper IUD. A randomized, double-blind clinical trial will be conducted with 72 participants randomly assigned to an experimental group (n=36) – active BBF and a control group (n=36) – simulated BBF. Patients will follow the IUD insertion protocol according to the guidelines of the Ministry of Health. Pain will be assessed at different times using the Visual Analogue Scale (VAS) during the insertion phases (Pozzi, Hysterometry and insertion) 5 and 15 minutes, 24 and 48 hours after IUD insertion . In addition, analgesic use and quality of life (WHOQOL-100) will be investigated over a 48-hour period, anxiety levels (GAD-7), satisfaction with the procedure immediately after insertion (15 minutes), and adverse and side effects over a 48-hour period. The duration of pain in hours from the moment of IUD insertion until its completion will also be assessed. And the success rate of the procedure. Statistical analysis will be performed using SPSS software version 24.0, with a significance level of 5% (p < 0.05). Data normality will be assessed using the Shapiro-Wilk test. Student's t-test or Mann-Whitney test will be used for continuous variables, and the chi-square or Fisher's exact test for categorical variables. Pain will be analyzed using the Friedman test, and logistic regression will assess associations between groups and adverse effects. Statistical analysis will be performed with a significance level of 5%. Data normality will be assessed using the Shapiro-Wilk test. For the analysis of pain (VAS) and variables such as anxiety and quality of life, the Friedman test will be applied. Analgesic use will be evaluated by ANOVA for repeated measures. Adverse effects will be analyzed by logistic regression. The time to cessation of abdominal discomfort will be estimated using Kaplan-Meier analysis, and IUD insertion success will be compared using the chi-square test.

**Keywords:** contraceptive methods, intrauterine device (IUD), pain control, photobiomodulation therapy, randomized clinical trial

**Abstract**

Unplanned pregnancy affects up to 65% of women in some regions of Brazil, increasing the risks of unsafe abortions and contributing to maternal mortality. The copper IUD is an effective and long-lasting contraceptive option, but its use is still limited in Brazil, covering only 4.4% of women of reproductive age. One of the main barriers is the pain associated with its insertion, which leads to fear and low adherence to the method. Since pain can be of visceral or somatic origin, traditional approaches such as anti-inflammatories and anesthetics have shown inconclusive results in reducing this discomfort. Photobiomodulation (PBM) has anti-inflammatory and analgesic effects, and has shown positive results in managing pelvic pain in other clinical contexts, such as labor. The objective of this study is to evaluate the efficacy of PBM as a preemptive analgesic method during the insertion of the T 380 copper IUD. A randomized, double-blind clinical trial will be conducted with 72 participants randomly allocated into an experimental group (n=36) – active PBM and a control group (n=36) – PBM simulation. Patients will follow the IUD insertion protocol according to the Ministry of Health guidelines. Pain will be assessed at different time points using the Visual Analog Scale (VAS) during the insertion phases (Pozzi, hysterometry, and IUD insertion), at 5 and 15 minutes, and at 24 and 48 hours after IUD insertion. Additionally, analgesic use and quality of life (WHOQOL-100) will be assessed over a 48-hour period, along with anxiety levels (GAD-7), satisfaction with the procedure immediately after insertion (15 minutes), and adverse and side effects within 48 hours. The duration of pain in hours from the moment of IUD insertion until its resolution will also be evaluated, as well as the success rate of the procedure. Statistical analysis will be performed using SPSS software version 24.0, with a significance level of 5% (p < 0.05). Data normality will be assessed using the Shapiro-Wilk test. Student's t-test or the Mann-Whitney test will be used for continuous variables, while the chi-square test or Fisher's exact test will be applied for categorical variables. Pain will be analyzed using the Friedman test, and logistic regression will evaluate associations between groups and adverse effects. Statistical analysis will be performed with a significance level of 5%. Data normality will be assessed using the Shapiro-Wilk test. For pain analysis (VAS) and variables such as anxiety and quality of life, the Friedman test will be applied. Analgesic use will be evaluated using repeated measures ANOVA. Adverse effects will be analyzed using logistic regression. The time to resolve abdominal discomfort will be estimated using Kaplan-Meier analysis, and IUD insertion success will be compared using the chi-square test.

**Keywords:** contraceptive methods, intrauterine device (IUD), pain management, photobiomodulation therapy, randomized clinical trial

**CONTEXTUALIZATION**

Unplanned pregnancy affects up to 65% of women ( Costa *et al* ., 2022 ) in some regions of the country, despite public policies aimed at guaranteeing reproductive rights. Unplanned pregnancy can lead to unsafe abortions and poor prenatal care, which are important causes of maternal mortality. Therefore, reproductive planning actions and guaranteed access to various contraceptive methods are necessary ( Costa *et al* ., 2022). Long-acting reversible contraceptive methods, such as the copper intrauterine device (IUD), help reduce the risk of unwanted pregnancy in the long term, especially among women with lower education and low socioeconomic status ( Neto *et al* ., 2021).

According to the 2019 National Health Survey, among women aged 15 to 49 who were still menstruating and had been sexually active in the last 12 months, 40.6% used birth control pills, 22.9% used some method of sterilization (17.3% tubal ligation and 5.6% vasectomy), and only 4.4% used an IUD, despite all its positive aspects compared to other methods ( Technical Manual for Health Professionals, 2018). Among the indications for the IUD, we can mention the desire for a long-term contraceptive method for women of reproductive age, including adolescence. It is an emergency contraceptive method, which can be inserted at any time during the cycle; it controls increased uterine bleeding (hormonal IUD); it controls dysmenorrhea (hormonal IUD); it is a contraceptive option for women with a personal and family history of thrombosis; It maintains pre-egg retrieval contraception (assisted reproduction) without worsening the outcome of the procedure (Ministry of Health, 2018).

Contraindications for IUDs include significant uterine cavity distortion, active pelvic inflammatory disease, known or suspected pregnancy, Wilson's disease or copper allergy, and abnormal uterine bleeding without a defined cause (Ministry of Health, 2018). For the insertion of an intrauterine device, the healthcare professional must know that the woman is not pregnant and that she does not present any signs or symptoms of pregnancy. Several criteria are used for this definition: insertion can be done during menstruation or up to 7 days after the start of normal menstruation; no sexual intercourse since the start of the last normal menstruation; correct and consistent use of a reliable method of contraception; less than 7 days after a spontaneous or induced abortion; within 4 weeks postpartum; or exclusive or near-exclusive breastfeeding, amenorrhea, and less than 6 months postpartum (Ministry of Health, 2018).

**IUD insertion**

The copper IUD is a contraceptive method that should be widely offered and implemented in Primary Health Care Units (UBS) and Specialized Medical Clinics (AME). However, in Brazil, there is a scarcity of studies that document and analyze the implementation of this method in health services. Given the apprehension surrounding the insertion and use of the IUD (Coleman et al., 2024), both on the part of women and health professionals, it is essential to promote research that deepens the understanding of the insertion procedures for this device in health services. These studies can contribute to demystifying the method, ensuring greater adherence to this form of contraception, which is effective and low-cost (Almeida T, et al., 2023; Barreto D. et al., 2020). This would allow for an expansion of its availability, guaranteeing greater access for the low-income population (Almeida T, et al., 2023; Barreto D. et al., 2020). One of the major barriers to IUD insertion is the pain during insertion and the resulting fear in patients of undergoing this procedure (Almeida et al., 2023; Lopes, et al., 2015).

Figure 1 - Technique for inserting an intrauterine device (IUD) on an outpatient basis.

**Innervation/ Spinal cord**

The spinal cord is responsible for the sensory innervation of the skin, muscles, joints, and viscera, and each of these groups is respectively called a dermatome, myotome, sclerotome , or viscerotome. Pain at the time of IUD insertion is... of two types: visceral and somatic.

Visceral pain is produced by dilation of the cervix, as afferent nerves are located between the muscle fibers of the cervix, and nerve impulses are transmitted to the spinal column by sensory nerves that are accompanied by sympathetic nerves. The dermatomes T10, T11, T12, and L1 (superior hypogastric plexus) are directly involved in this process. pain perception (Erdoğan *et al* ., 2023).

Somatic pain results from the stretching of the pelvic floor muscles, vagina, and perineum, and the impulses Painful signals are conducted by the pudendal nerves, specifically the dermatomes S2, S3, S4, the most important in pain perception (Erdoğan *et al* ., 2023).

Neuroanatomy shows that the nerves of the vagina and uterus are derived from the uterovaginal nerve plexus, which is one of the pelvic plexuses that extend from the inferior hypogastric plexus to the pelvic viscera. Sympathetic, parasympathetic, and visceral afferent fibers traverse this plexus. (Moore, 2014 )

Sympathetic innervation originates in the lower thoracic segments (T10) of the spinal cord and travels along the lumbar splanchnic nerves and the series of intermesenteric-hypogastric-pelvic plexuses. Parasympathetic innervation originates in the S2–S4 segments of the spinal cord and travels along the pelvic splanchnic nerves to the inferior hypogastric-uterovaginal plexus. Visceral afferent fibers that conduct intraperitoneal pain impulses from the fundus and body of the uterus (above the pelvic pain line) follow retrograde sympathetic innervation to reach the cell bodies in the sensory ganglia of the lower thoracic-upper lumbar spinal nerves. The afferent fibers that conduct pain impulses from the cervix and vagina (below the pelvic pain line) subperitoneally follow the parasympathetic fibers retrogradely through the uterovaginal and inferior hypogastric plexuses and the pelvic splanchnic nerves to reach the cell bodies in the sensory ganglia of spinal nerves S2–S4. The two different pathways followed by visceral pain fibers are clinically important because they provide various types of anesthesia during childbirth, pelvic procedures, and the control of chronic pain in the pelvic region. All visceral afferent fibers from the uterus and vagina not related to pain (those that conduct unconscious sensations) also follow the latter pathway. (Moore, 2014 )

**Pain relief methods**

Several studies have been conducted to try to control and reduce discomfort during IUD insertion (Lopes *et al* ., 2015; Neto ED da S et al., 2021; Almeida T. et al., 2023; Erdoğan P et al., 2023 ), but always with inconclusive results, whether with the use of non-steroidal anti-inflammatory drugs (NSAIDs) (Lopes *et al* ., 2015; Neto ED da S et al., 2021; Almeida T. et al., 2023; Erdoğan P et al., 2023 ), the use of local anesthetics (Lopes *et al* ., 2015), or cervical preparation (Lopes *et al* ., 2015; Neto ED da S et al., 2021; Almeida T. et al., 2023; Erdoğan P et al., 2023). ).

In a review by Cohrane (Lopes *et al* ., 2015), it was demonstrated that 2% lidocaine gel, misoprostol, and most NSAIDs (non-steroidal anti-inflammatory drugs) did not help reduce pain and that these interventions are ineffective, not requiring further research on the subject. Some formulations of lidocaine, tramadol, and naproxen showed some effect in reducing pain related to IUD insertion in some specific groups. Most of the evidence of efficacy was of moderate quality, coming from isolated trials.

Given the lack of scientific evidence on the effectiveness of local analgesia in reducing pain during IUD insertion, we sought other forms of analgesia for the control of acute and chronic pelvic pain, as well as during labor. Studies were found showing that paravertebral nerve blocks between T10 and S4 with anesthetics, distilled water, and photobiomodulation show some level of results ( Traverzim *et al* ., 2018; Almeida *et al* ., 2023; Neto *et al* ., 2021). Some paravertebral stimulation methods have been effective in controlling pelvic pain, especially during labor. Among them, we can mention Transcutaneous Electrical Nerve Stimulation (TENS): TENS has been widely used for analgesia during childbirth. Although the exact mechanism is not yet fully understood (Njogu et al., 2021), studies confirm its effectiveness and safety. The application of high-frequency TENS (80 to 100 Hz) and a pulse width of 350 microseconds, with two pairs of electrodes positioned between the paravertebral levels T10-L1 and S2-S4 during the active phase of labor, resulted in a significant reduction in pain, as assessed by the Visual Analogue Scale (VAS) (Soares et al., 2022).

As an anesthetic method, we also have the anesthetic block. The paraspinal anesthetic block consists of anesthetizing the spinal segment between T10 and L2, responsible for the innervation of the pelvic viscera. This method provides rapid relief for acute pelvic pain, but is not indicated for the management of chronic pain (Rosa et al., 2013).

In a study conducted in 2018 at the Mandaqui Hospital Complex, in collaboration with the Biophotonics service of Uninove, irradiation with red and infrared LEDs in the paravertebral region between T10 and S4 proved to be effective in analgesia during labor, promoting comfort and reducing pelvic pain (Traverzim et al., 2018).

**Choosing a comparator**

Manipulation of the cervix and passage of the device through the internal os can be uncomfortable for some women. The article by Tabatabaei et al. (2024) discusses pain associated with the insertion of intrauterine devices (IUDs) as a factor to be carefully considered to avoid complications. Mild to moderate pain is considered normal during insertion. However, severe or persistent pain is indicative of complications such as uterine perforation or visceral damage. Healthcare professionals should not underestimate the pain reported by patients or fail to perform the necessary complementary examinations to verify the correct position of the IUD, which can result in late diagnoses and unnecessary reinsertions (Tabatabaei et al. 2024).

Deeper anesthesia is contraindicated, as a low level of pain is desirable, as described above. Some strategies can be used, although they have not been scientifically proven effective, such as the administration of oral nonsteroidal anti-inflammatory drugs (NSAIDs) before IUD insertion. The control group in IUD insertion studies may consist of a placebo, no intervention, or another active intervention (Lopes, 2015).

**Photobiomodulation in pain management**

Recent studies have demonstrated the effectiveness of photobiomodulation in the treatment of lower back pain, using different wavelengths. Lin et al. (2020) observed good results with irradiation of the thoracolumbar region, using wavelengths between 630 and 850 nm. In another study, Tomazoni et al. (2020) employed 490 nm with correction to 570 nm and found changes in microcirculation and in the control of the release of inflammatory interleukins.

Additionally, the application of 905 nm for 3 minutes, covering a larger area (T11 to S1), was effective for the management of chronic low back pain (Tomazoni et al., 2017). In another study, Traverzim (2018) used irradiation between T10 and S4 for analgesia during labor, applying 1.5 J of energy at red and infrared wavelengths, with promising results.

Given the promising results observed with the use of non-pharmacological therapies for IUD insertion ( Gemzell-Danielsson, 2019 ) for analgesia during childbirth (Traverzim, 2018), and considering that the Ministry of Health recommends intrauterine IUD insertion without the use of any analgesic, it becomes essential to investigate ways to minimize the pain reported by women during and after the insertion of the device. Further studies are needed to explore alternatives that can reduce this discomfort, encouraging more women to adopt this contraceptive method, which is effective and low-cost, promoting a positive impact on Brazilian public health.

**Justification**

Unplanned pregnancy affects up to 65% of women in some regions of Brazil (Costa et al., 2022), despite public policies aimed at guaranteeing reproductive rights. This scenario increases the risk of unsafe abortions and compromises prenatal care, constituting significant causes of maternal mortality. Therefore, it is essential to implement reproductive planning actions that ensure access to effective contraceptive methods (Ministry of Health 2018).

The copper intrauterine device (IUD) is a long-acting reversible contraceptive method that stands out for significantly reducing the risk of unwanted pregnancy, especially in women with lower levels of education and low socioeconomic status (Neto et al., 2021). However, pain during and after IUD insertion is a factor that discourages many women from choosing this method, compromising adherence and continued use.

Healthcare professionals still need better interventions than those generally used. According to a 2015 Cochrane review (Lopes, 2015), almost all trials used intrauterine contraceptives. Most of the efficacy evidence was considered to be of moderate quality, as it was based on isolated studies. 2% lidocaine gel, misoprostol, and most NSAIDs did not reduce pain. Some formulations of lidocaine, tramadol, and naproxen showed an effect in reducing pain related to IUD insertion in specific groups. The authors conclude that interventions without effect do not require further investigation.

In this context, it becomes necessary to investigate alternatives to reduce the discomfort associated with IUD insertion, promoting greater acceptance and adherence to the method. Photobiomodulation (PBM), due to its proven anti-inflammatory and analgesic action, emerges as a promising alternative. By irradiating the nerve endings in the thoracolumbar region, PBM can inhibit the propagation of pain to somatic and visceral organs, providing a more comfortable procedure and faster recovery.

Thus, this study is justified by the need to evaluate whether the use of FBM during the insertion of the Cu-T 380 IUD in a single session can contribute to the reduction of intra- and post-procedure pain, promoting greater comfort and well-being for women and, consequently, encouraging the adoption of a safe and low-cost contraceptive method, with a positive impact on Brazilian public health.

**Experimental hypothesis**

The use of FBM (Functional Biomechanical Monitoring) can reduce the perception of pain during the insertion of the T 380 copper IUD for contraception.

## **OBJECTIVES**

### **2.1. General objective**

The aim of this study is to evaluate whether preventively applied photobiomodulation alters pain perception during insertion of a T380 copper IUD for contraception in women without comorbidities.

### **2.2. Specific objectives**

## Pain assessment (VAS) during the IUD insertion phases (Pozzi, Hysterometry and IUD insertion) 5 and 15 minutes, 24 and 48 hours after IUD insertion.

## Quantity of medication (paracetamol) ingested by the patient during a 48-hour period.

## Assessment of patients' anxiety using the Generalized Anxiety Disorder 7 (GAD-7) instrument.

## Assessment of patients' quality of life using the WHOQOL-Pain instrument.

## Patient satisfaction through a structured questionnaire (Lopes, 2015)

## Time for abdominal discomfort to subside

## Adverse effects (e.g., bleeding, fainting, allergy)

## Side effects (cramps, chills, numbness of the tongue)

## Failure to insert the IUD.

## **MATERIALS AND METHODS**

This is a single-center, randomized, double-blind, controlled, parallel-group, superiority clinical trial designed according to the SPIRIT *Statement criteria.* ( <https://www.spirit-statement.org/>)

The project will be submitted to the Research Ethics Committee (CEP) of the Mandaqui Hospital Complex in the city of São Paulo . Any incident or alteration during the study will be reported and clarified to the CEP and in future publications of this study. After a verbal explanation by the principal investigator, and a written explanation of the study, participants who agree to participate will sign the Informed Consent Form (TCLE). Participants who wish to receive the research data will provide their *email address* in the TCLE, and the complete article will be provided as soon as it is published. The treatments will be performed at the Mandaqui Hospital Complex, located in the North Zone of the city of São Paulo, Brazil, from November 2024 to April 2026 by a gynecologist with more than 10 years of experience. The project will be registered on the *Clinicaltrials platform* ( <https://clinicaltrials.gov/>).

**Calibration/training**

There will be only one examiner who will evaluate 5 women, who will not be part of the study. Clinical pain assessments will be performed exactly as proposed in this work. The principal investigator is a gynecologist with over 10 years of experience in the field and will perform all IUD insertions and postoperative evaluations. In this work, the principal investigator will be trained to assess the following outcomes: anxiety, using the Generalized Anxiety Disorder 7 (GAD-7) questionnaire; quality of life, using the WHOQOL-Pain instrument; and patient satisfaction, based on a structured questionnaire, as per the study by Lopes (2015).

**Sample size calculation**

The total sample size will be 72 patients per group. This value was calculated to provide a power of 80% (α = 0.05) and an effect size of 0.14. To determine the number of patients in each group, a sample size calculation was performed using G*Power 3.1.9.7 software. The sample was calculated for 2 groups. Eight measurements will be taken during the IUD insertion phases (Pozzi, Hysterometry, and IUD insertion) 5 and 15 minutes, 24 and 48 hours after IUD insertion .


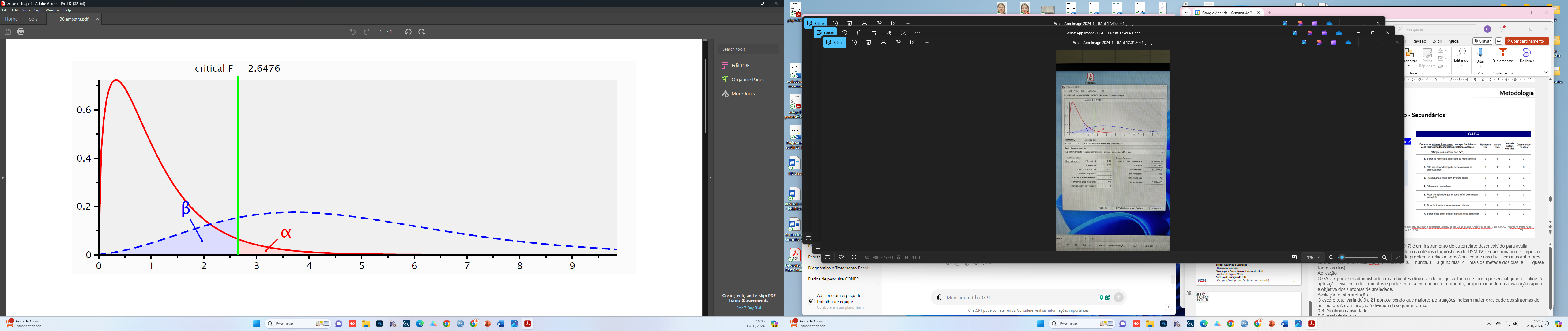


Source: G*Power 3.1.9.7 software.

### **3.1. Sample description**

Women of reproductive age referred to the hospital for contraception will be selected from the Primary Health Care Units (UBSs) in the North Zone of São Paulo or from the gynecological emergency room of the Mandaqui Hospital Complex.

**Inclusion/Exclusion Criteria**

Inclusion criteria:

- Participants aged 18-50,
- Female,
- Without preference for race or socioeconomic level,
- Nulliparous or multiparous

Exclusion criteria:

- Known or suspected pregnancy,
- Diagnosed chronic pain,
- Active local infection,
- Any pain medication taken in the last 12 hours,
- Known contraindications for IUD insertion (significant distortion of the uterine cavity, active pelvic inflammatory disease, Wilson's disease).
- Copper allergy (Ministry of Health, 2018)
- Abnormal uterine bleeding without a defined cause (Ministry of Health, 2018).
- With any alteration in the lumbar region, such as: active neoplasms, established osteomyelitis, any pre-existing deep tissue lesion, with necrosis or infection,
- With a history of photosensitivity.
- Patients with a BMI ≥25 kg/m2 ^(^ WHO, 2000)

**Study termination criteria**

There are no plans to interrupt or modify the interventions allocated to a given trial participant because the control group will receive the treatment recommended by the Ministry of Health, based on a Cochrane systematic review (Lopes et al., 2015) and recommended by the intrauterine device (IUD) manufacturer. To date, there is no evidence of treatment for analgesia during IUD insertion (Lopes et al., 2015). In the experimental group, a procedure will be performed that does not cause any adverse or side effects; there are reports of analgesia during labor ( Traverzim et al., 2018). Therefore, as the study poses no risk to patients, there is no basis for interrupting the study.

**Recruitment**

Recruitment will be on a walk-in basis, with participants referred to the hospital for contraception from the Primary Health Care Units (UBSs) in the North Zone of São Paulo or from the gynecological emergency room of the Mandaqui Hospital Complex.

**Application of anamnesis and collection of risk factors.**

A medical history ( ANNEX 1) will be taken from all patients. In addition to questions related to general health, demographic data (age, gender, marital status, occupation, educational level, salary), medical history data (chief complaint, current illness status, medical history, medications in use) , presence of smoking or alcoholism, use of systemic antibiotic therapy in the last 12 (twelve) months, hospitalization or infection (including skin infection) in the last 12 (twelve) months, whether an IUD has been used previously, and contraceptive methods usually used will be collected.

This questionnaire will allow us to correlate clinical, demographic, and epidemiological risk factors that may be related to the results obtained in this population.

**Allocation (sequence generation, allocation hiding mechanism, implementation)**

The treatment performed immediately after IUD insertion will be randomized; it may be photobiomodulation or photobiomodulation simulation. An online random sequence generator program ( <https://www.sealedenvelope.com/>) will be used, and the randomization option will be selected in blocks of 2 treatments at a 1:1 ratio, meaning the same number of patients in both groups (n=36). Since the study has a total sample of 72 patients, 12 blocks of 6 patients will be conducted. Opaque envelopes will be identified with sequential numbers (from 1 to 72), and the information of the corresponding experimental group will be inserted inside, according to the generated random sequence. The envelopes will be sealed and kept in a safe place until the IUD insertion. Only the healthcare professional will know the nature of the treatments. The envelope will be resealed and kept with the patient's medical record, only to be revealed at the end of the statistical analysis. The generation of the random sequence and the preparation of the envelopes will be carried out by a person not directly involved in the study.

All participants will undergo the same insertion protocol with the same professional, as described in the "IUD Insertion Protocol" section. Immediately after insertion is complete, the researcher responsible for administering the FBM will remove and open one envelope (without altering the numerical sequence of the other envelopes) and perform the indicated procedure or its simulation. Only this researcher will know the nature of the treatments.

The researcher responsible for the study will be responsible for storing (digital platforms) and tabulating the information. The statistician will be responsible for analyzing the data obtained at the end of the study and will be blinded to the interventions. These same envelopes will contain the participants' forms (anamnesis questionnaire, demographic data and risk factors, and the informed consent form) and will be kept on file.

**Study blinding (masking)**

Only the researcher responsible for administering the treatments (who will open the randomization envelopes) will know which treatment is assigned to each patient. The patient will be blinded due to the similarity of the treatments (LED on/LED off). The researcher responsible for collecting the outcomes will be blinded to the interventions performed because they will not be in the room at the time of treatment application. The identification of each group will only be revealed after statistical analysis of the data for all those involved in the study by this researcher. Therefore, the researcher responsible for data collection and the statistician will also be blinded to the treatments assigned to the groups. During the trial, the study's unmasking is not planned because no adverse events are expected with the use of FBM.

**IUD insertion protocol according to the recommendations of the Ministry of Health.**

Before the procedure, the steps will be explained again to the patient to reduce anxiety and promote greater relaxation during IUD insertion. A bimanual pelvic exam will be performed to assess the size, position, and mobility of the uterus. Infection prevention measures will be taken, using sterile gloves and cleaning the cervix with a chlorhexidine hydrochloride-based antiseptic. Next, the anterior lip of the cervix will be gently pinched with Pozzi forceps for stabilization during insertion. The hysterometer will be introduced slowly and carefully to determine uterine depth and angulation, minimizing the risk of perforation. During IUD preparation, instruments and gloves will be kept sterile, and the device will be assembled according to the manufacturer's instructions, keeping the arms in a horizontal position during insertion. The IUD will be inserted using the retractable technique: the insertion tube will be introduced to the uterine fundus and then partially withdrawn, keeping the plunger fixed to release the IUD strings. After a few seconds, the plunger and guide tube will be removed. The IUD strings will be cut, leaving them 2 to 3 cm long in relation to the cervix, and the patient will remain lying down for about 15 minutes after the procedure to reduce discomfort. After insertion, the patient will be monitored to assess her well-being and possible vasovagal reactions (such as sweating, vomiting, or fainting), which are rare and self-limiting. The length of the IUD strings in relation to the cervix will be recorded in the medical record, and the patient will be instructed to return for a follow-up appointment in 30 days to check the device's position and assess adaptation.

### **Group composition**

The 72 participants will be allocated into the Control and experimental groups as follows:

**Control Group – FBM Simulation (n=36 participants):** All participants will undergo the same conventional IUD insertion procedure as described previously. Participants will receive a simulation of FBM and will be treated identically to the Experimental group. The researcher responsible for applying FBM will simulate the irradiations by positioning the device in the same location described for the FBM group; however, the equipment will remain switched off. To prevent the participant from identifying which group she belongs to, the activation sound of the equipment (beep) will be recorded and played at the time of application.

**Experimental Group – FBM (n=36 participants):** All participants will undergo the same procedure. For irradiation, a Sportlux® brand LED panel (Brazil, SP) (Figure 1) with the following specifications will be used. The equipment description, dosimetric parameters, and number of FBM applications are described in Table 1.

**Table 1:** Dosimetric parameters used for preventive FBM.

| **TECHNICAL PARAMETERS** | **Sportllux®** |
| --- | --- |
| Light source | LED |
| Application technique | Contact |
| Wave-length | 132 LEDs with 660nm |
|  | 132 LEDs with 850nm |
| Spectral band | 20 nm |
| Beam area at the target | 0.5 cm² |
| Irradiation time | 20 min |
| Average power of each LED | 8mW |
| Irradiance | 16 mW/cm² |
| Application time | 10 minutes |
| LED Energy | 4.8J |
| Radiant exposure | 9.6 J/cm² |
| Angle of light emission | 120° |

nm- nanometer, W- Watt, J- Joule,


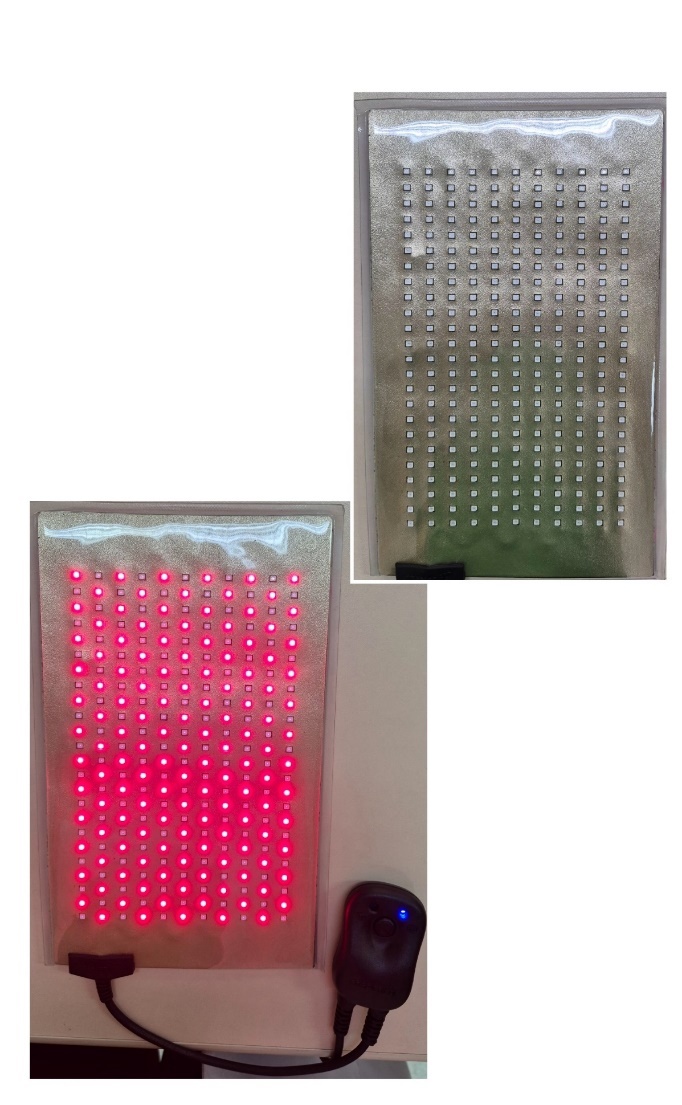

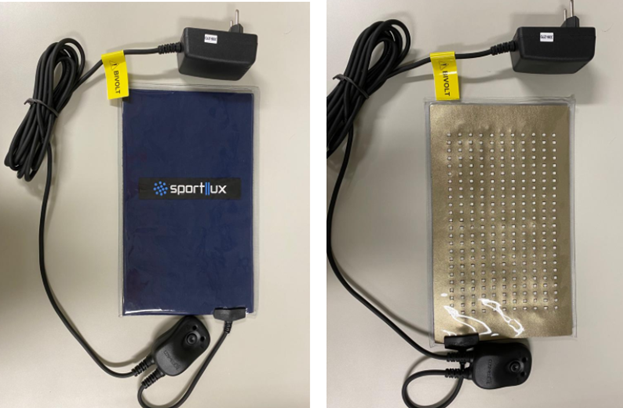

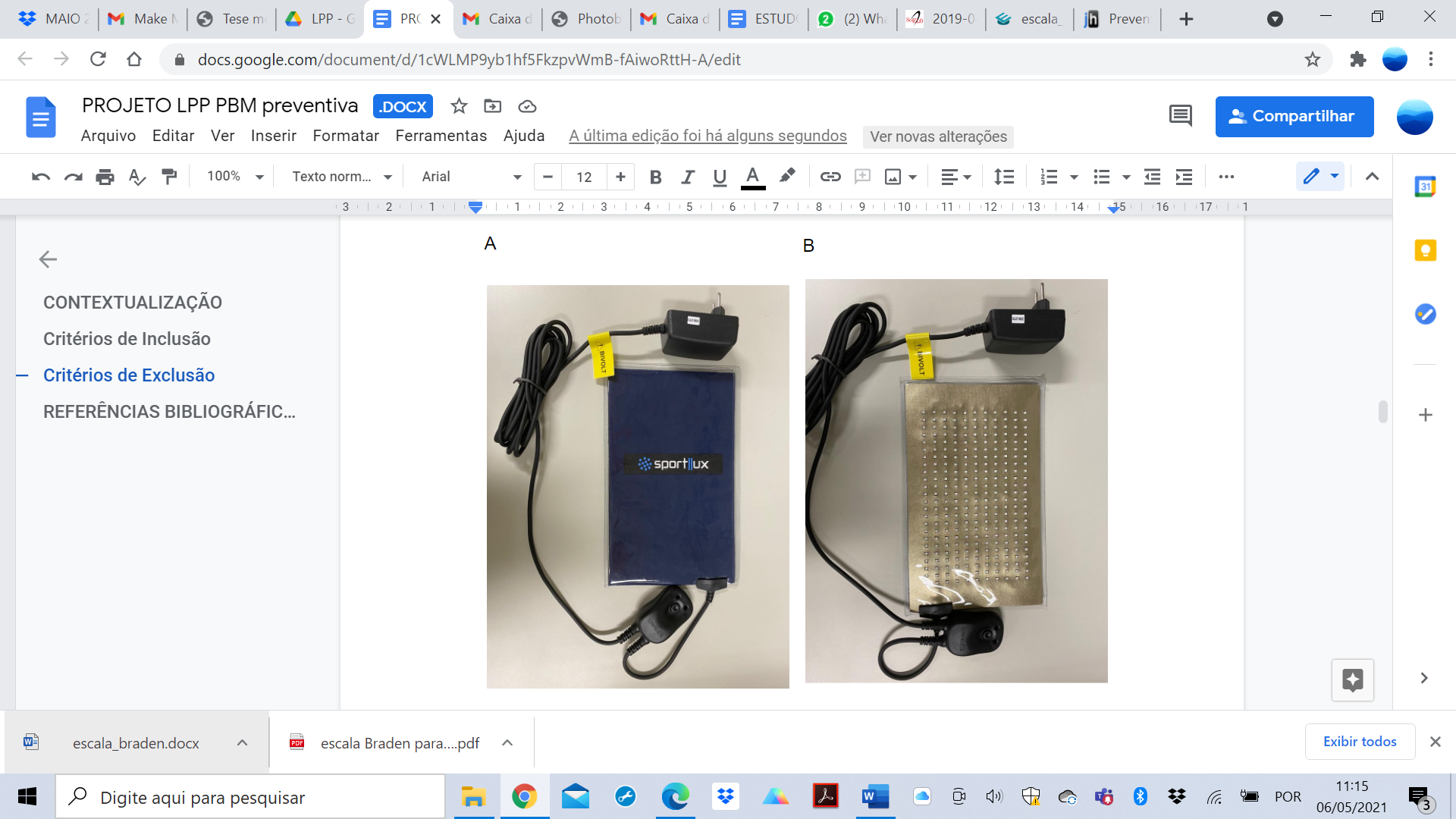


C

B

A

Figure 1: Sportlux® brand LED panel used for preventive irradiation during IUD insertion. Figures A and B: Panel off. Figure C: Panel on.

The irradiated region will cover the lumbar and thoracic spine, more specifically from T10 to L4, using the LED panel in a vertical direction.

Figure 2. Study flowchart

Recrutamento

Treinamento dos pesquisadores

Seleção dos participantes conforme critérios de inclusão

Convite para participar e assinatura do termo de consentimento livre e esclarecido (TCLE)

(Estimativa de 10% de evasão do estudo)

Aleatorização

*Baseline*  (T0)

EVA, entrega da cartela de analgésicos aplicação dos questionários de satisfação e qualidade de vida e ansiedade

*Baseline*  (T0)

EVA, entrega da cartela de analgésicos aplicação dos questionários de satisfação e qualidade de vida e ansiedade

Inserção do DIU CU T 380 + FBM

Inserção do DIU CU T 380 + FBM placebo

(n=xxx)

1^o^ avaliação (T1=6h)

EVA, contagem de analgésicos aplicação dos questionários de satisfação e qualidade de vida e ansiedade

1^o^ avaliação (T1=6h)

EVA, contagem de analgésicos aplicação dos questionários de satisfação e qualidade de vida e ansiedade

2^o^ dia de avaliação (T2=24h)

EVA, contagem de analgésicos aplicação dos questionários de satisfação e qualidade de vida e ansiedade

2^o^ dia de avaliação (T2=24h)

EVA, contagem de analgésicos aplicação dos questionários de satisfação e qualidade de vida e ansiedade

3^o^ dia de avaliação (T3=48h)

EVA, contagem de analgésicos aplicação dos questionários de satisfação e qualidade de vida e ansiedade

3^o^ dia de avaliação (T3=48h)

EVA, contagem de analgésicos aplicação dos questionários de satisfação e qualidade de vida e ansiedade

3^o^ dia de avaliação (T3=48h)

EVA, contagem de analgésicos aplicação dos questionários de satisfação e qualidade de vida e ansiedade

3^o^ dia de avaliação (T3=48h)

EVA, contagem de analgésicos aplicação dos questionários de satisfação e qualidade de vida e ansiedade

**Study outcome variables**

- Pain – Assessment using the Visual Analogue Scale (VAS) at baseline, during the IUD insertion phases (Pozzi, Hysterometry and IUD insertion) 5 and 15 minutes, 24 and 48 hours after IUD insertion.
- Description of pain assessment – Free report
- Amount of analgesics ingested - (paracetamol) ingested by the patient after insertion of the T 380 copper IUD in the period from baseline up to 48 hours after insertion.
- Assessment of anxiety in patients undergoing insertion of a T380 copper IUD for contraception using the *Generalized Anxiety Disorder 7* ( GAD-7 ) instrument (Bártolo A, 2017) in the period immediately after insertion (15 minutes).
- Assessment of quality of life using the WHOQOL-Pain instrument in patients undergoing insertion of a T380 copper IUD for contraception **(Pedroso et al 2015)** during the period from baseline up to 48 hours after insertion.
- Patient satisfaction after insertion of the T380 copper IUD for contraception was assessed using structured questions (Lopes, et al 2015) in the period immediately following insertion (15 minutes).
- Time required for abdominal discomfort (menstrual cramps) to cease, measured in days, from baseline to 48 hours after insertion.
- Ultrasound to verify successful insertion. If IUD insertion fails, it will be evaluated dichotomously (success/failure) in the period from baseline up to 48 hours after insertion.
- Adverse effects (Uterine perforation, IUD displacement, abdominal pain and increased vaginal bleeding, allergy). An open-ended question will be asked so that the patient can openly answer about the adverse effects, and then they will be listed by name so that she can remember any effect she may have forgotten to report.
- Side effects (cramps, mild pain, slight bleeding, numbness of the tongue). An open-ended question will be asked so that the patient can openly answer about the side effects, and then they will be listed by name so that she can remember any effect she may have forgotten to mention.

**Pain - Visual Analogue Scale (VAS)**

Pain will be assessed using the Visual Analogue Scale (VAS) as shown in the diagram below. One extreme indicates '0', and the other '10', meaning respectively 'no pain' and 'unbearable pain'. This scale will be the same for all participants. Instructions on marking will always be given by the same operator. The pain value will be recorded using scores. This analysis will occur at all consultations ( during the IUD insertion phases - Pozzi, Hysterometry, and IUD insertion - 5 and 15 minutes, 24 and 48 hours after IUD insertion ). Participants will be asked about spontaneous pain at three distinct moments during insertion: pain during the Pozzi test (pinch), pain during hysterometry (uterine length measurement), and pain during the insertion itself. All patients will be reminded by daily phone calls to record their outcomes as recommended (Qamrudin 2014).

### *Cervical Clamping:* Gently clamp the anterior lip of the cervix with Pozzi forceps for stabilization during insertion.

### *Hysterometry:* Introduce the hysterometer slowly and carefully to determine uterine depth and angulation, minimizing the risk of uterine perforation.

- *Insertion into the uterine cavity:*

**Description of pain assessment – Free report**

Immediately after IUD insertion, patients will be asked to report on their experience with the procedure, and their account will be recorded in the participant's own words for qualitative evaluation.

**Amount of analgesics ingested - (paracetamol) ingested by the patient after insertion of the T 380 copper IUD at baseline, during insertion, 5 min, 15 min, 24 h, and 48 h after insertion.**

The amount of analgesics ingested will be evaluated as proposed by Bauer 2013. At the beginning of the research, each participant will be given a blister pack of paracetamol® (a drug with a purely analgesic effect) (Jóźwiak-Bebenista, 2014), which must be kept until the end of the study and its use will only be permitted in case of pain. At the end of the experiment, the number of tablets will be evaluated as another parameter for measuring pain. The analgesic used will be 500mg paracetamol. It should only be used if there is pain. The recommended dose, if necessary, will be 1 tablet every 6 hours. The participant will be asked to record in writing the amount of analgesics ingested, the day, and the time. A procedure will be carried out to monitor participant adherence (participants will be asked to bring the blister pack of analgesics to the consultation for verification). Medication use will be monitored from baseline until 48 hours after administration.

**Assessment of anxiety in patients undergoing insertion of a T380 copper IUD for contraception using the *Generalized Anxiety Disorder 7-item* (GAD-7) instrument at baseline and 5 minutes after the end of the procedure ( Bártolo A, 2017).**

The *Generalized Anxiety Disorder 7* (GAD-7) is a self-report instrument developed to assess symptoms of generalized anxiety, based on the DSM-IV diagnostic criteria. The questionnaire consists of seven items that measure the frequency of anxiety-related problems in the previous two weeks, based on a 4-point Likert scale (0 = never, 1 = some days, 2 = more than half the days, and 3 = almost every day). The GAD-7 can be administered in clinical and research settings, both in person and online. The application takes approximately 5 minutes and can be done in a single session, providing a quick and objective assessment of anxiety symptoms.

The total score ranges from 0 to 21 points, with higher scores indicating greater severity of anxiety symptoms. The classification is divided as follows:

- 0-4: No anxiety
- 5-9: Mild anxiety
- 10-14: Moderate anxiety
- 15-21: Severe anxiety

The GAD-7 has good validity and reliability, with extensive use in clinical and non-clinical populations. The questionnaire structure can be analyzed using a unifactorial or bifactorial model (somatic and cognitive-emotional items), depending on the application context.

**Assessment of the quality of life of patients undergoing insertion of a T380 copper IUD for contraception using the** WHOQOL-Pain instrument. **baseline and 48 hours after the procedure is completed.**

The WHOQOL-Pain is an add-on module to the WHOQOL-100 instrument, developed specifically to assess the quality of life in people living with chronic pain. After a preliminary study, ten new facets related to pain and discomfort were identified. Of these, four facets were selected to compose the final version of the instrument: **Pain relief , Anger and frustration , Vulnerability, fear and worry, and Uncertainty (Pedroso et al., 2015) .** Each facet consists of four questions that address different dimensions of the pain experience. The responses follow a five-point Likert scale, measuring aspects such as intensity, capacity, evaluation, and frequency. The scores are transformed into a scale of 0 to 100, allowing for a quantitative interpretation of pain levels and their impact on quality of life. The WHOQOL-Pain questions are not integrated into the WHOQOL-100, being applied separately at the end of the main questionnaire. This structure facilitates the individual analysis of pain-related facets, allowing for a specific and detailed assessment of chronic physical pain experiences.

**Patient satisfaction after insertion of the T380 copper IUD for contraception was assessed through guiding questions (Lopes, et al 2015) after the procedure (15 minutes).**

- the onboarding experience unpleasant?
- Would you do it again in the future?
- Would you recommend it to a friend?

**Time period for abdominal discomfort to cease after FBM, measured in hours.**

The time from the onset of colic until the moment it ends and there is no more pain will be recorded. This outcome will be measured in hours.

**Ultrasound to verify successful placement.**

If the IUD insertion fails, it will be evaluated dichotomously (success/failure).

**Adverse effects** **(Uterine perforation, IUD displacement, abdominal pain and increased vaginal bleeding, allergy).**

An open-ended question will be asked so that the patient can openly answer about the adverse effects, and then they will be listed by name so that she can remember any effect she may have forgotten to report.

**Side effects (cramps, mild pain, slight bleeding, numbness of the tongue).**

An open-ended question will be asked so that the patient can openly answer about the side effects, and then they will be listed by name so that she can remember any effect she may have forgotten to mention.

**Ethical aspects**

This study ensures compliance with applicable ethical and regulatory guidelines. No modifications to the research protocol are anticipated, but should any occur, they will be promptly communicated to stakeholders, including investigators, the ethics committee (amendment submission), participants, and clinical trial registries.

Free and informed consent will be obtained by a trained researcher, who will provide participants with detailed information about the objectives, procedures, risks, and benefits of the study, using a consent form written in clear and accessible language. No biological materials will be collected or stored.

The collection, sharing, and storage of participants' personal information will follow strict data protection protocols to ensure confidentiality before, during, and after the trial. All data will be anonymized and securely stored to prevent unauthorized access. There are no financial or other conflicts of interest. Only the principal investigator will have access to the final study dataset.

The principal investigator agrees to disseminate the study results to participants, healthcare professionals, and the general public through publications, presentations at conferences, and respecting any previously agreed-upon publication restrictions.

**Statistical analysis**

Statistical analysis will be performed using the *Statistical Package for the Social Sciences* (SPSS) version 24.0 or equivalent software. The significance level will be set at 5% (p < 0.05) for all tests. Data normality will be assessed using the Shapiro-Wilk test. Continuous variables will be described using mean and standard deviation, or median and interquartile range, depending on the data distribution. Categorical variables will be presented as absolute and relative frequencies.

For comparison between the experimental group (active FBM) and the control group (FBM simulation), the Mann-Whitney test will be used for continuous variables with a non-normal distribution. Categorical variables will be compared using the chi-square test or Fisher's exact test, depending on whether the minimum expected value is less than 5.

The analysis of variables related to pain (intensity measured by the Visual Analog Scale – VAS) at different times (baseline, during insertion, 5 and 15 minutes, 24 and 48 hours after the procedure) will be performed using the Friedman test or a generalized linear model, adjusting for possible covariates.

To assess anxiety (GAD-7), quality of life (WHOQOL-100), and satisfaction with the procedure, the Mann-Whitney test will be performed for comparison between the groups at different assessment times.

The use of post-procedure analgesics will be compared between groups using repeated measures analysis of variance (ANOVA), considering the number of tablets ingested at each time point (24h and 48h). Additionally, to verify the association between the groups and the occurrence of adverse and side effects, logistic regression will be performed, considering the group as the predictor variable and adverse effects as the response variable.

Finally, a Kaplan-Meier analysis will be performed to estimate the time required for the cessation of abdominal discomfort (cramps), and the success of IUD insertion will be analyzed by comparing proportions between the groups using a chi-square test.

**Bibliographic references**

Almeida, Tiago & Mata, André & Leitão, Cláudia & Basto, Daniela & Ferreira, Mariana. (2023). Management of pain related to the intrauterine device insertion: what is the evidence?. 17. 287-298.

Barreto D da S, Maia DS, Gonçalves RD, Soares R de S. Intrauterine Device in Primary Health Care: an integrative review. Rev Bras Med Fam Comunidade [Internet]. May 30, 2021 [cited July 15, 2024];16(43):2821. Available from: <https://rbmfc.org.br/rbmfc/article/view/2821>

Bártolo A, Monteiro S, Pereira A. Factor structure and construct validity of the Generalized Anxiety Disorder 7-item (GAD-7) among Portuguese college students. Public Health Cad. 2017 Sep 28;33(9):e00212716. doi: 10.1590/0102-311X00212716. PMID: 28977285.

Brazil. Ministry of Health. Secretariat of Health Care. Department of Strategic Programmatic Actions. [Technical Manual for Health Professionals: Copper IUD TCu 380A](https://portaldeboaspraticas.iff.fiocruz.br/wp-content/uploads/2018/12/manual_diu_08_2018.pdf) / Ministry of Health, Secretariat of Health Care. Department of Strategic Programmatic Actions. – Brasília: Ministry of Health, 2018.

Coleman K, Carter C. Pain control during intrauterine device insertion: Transcutaneous electrical nerve stimulation. J Am Assoc Nurse Pract. 2024 Oct 1;36(10):594-596. doi: 10.1097/JXX.0000000000001031. PMID: 38787344.

Costa, Ana & Lanhoso, António & Capela, Eunice & Neves, Joaquim & Reis, José & Silva, Daniel. (2022). Pain management in office gynecological procedures. 16. 264-272.

Erdoğan P, Yardımcı H. Analgesic effects of LI4 acupuncture during intrauterine device insertion: a randomized controlled clinical trial. Arch Gynecol Obstet. 2023 Oct;308(4):1361-1368. doi: 10.1007/s00404-023-07106-5. Epub 2023 Jul 19. PMID: 37466690.

Durnin JV, Womersley J. Body fat assessed from total body density and its estimation from skinfold thickness: measurements on 481 men and women aged from 16 to 72 years. Br J Nutr. 1974 Jul;32(1):77-97. doi: 10.1079/bjn19740060. PMID: 4843734.

Gemzell-Danielsson K, Jensen JT, Monteiro I, Peers T, Rodriguez M, Di Spiezio Sardo A, Bahamondes L. Interventions for the prevention of pain associated with the placement of intrauterine contraceptives: An updated review. Acta Obstet Gynecol Scand. 2019 Dec;98(12):1500-1513. doi: 10.1111/aogs.13662. Epub 2019 Jun 27. PMID: 31112295; PMCID: PMC6900125.

Lin YP, Su YH, Chin SF, Chou YC, Chia WT. Light-emitting diode photobiomodulation therapy for non-specific low back pain in working nurses: A single-center, double-blind, prospective, randomized controlled trial. Medicine. 7 Aug 2020;99(32):e21611.

Lopez LM, Bernholc A, Zeng Y, Allen RH, Bartz D, O'Brien PA, Hubacher D. Interventions for pain with intrauterine device insertion. Cochrane Database Syst Rev. 2015 Jul 29;2015(7):CD007373. doi: 10.1002/14651858.CD007373.pub3. PMID: 26222246; PMCID: PMC9580985.

Technical Manual for Healthcare Professionals: Copper IUD TCu 380A / Ministry of Health, Secretariat of Health Care. Department of Strategic Programmatic Actions. – Brasília: Ministry of Health, 2018.

Mc Mahon SB Koltzenburg M (eds). Wall and Melzack's Textbook of pain 5 ed Elsevier, London, United Kingdom, 2006

Moore AFD. Clinically Oriented Anatomy. Guanabara Koogan Ltda Publisher; 2014, Jan 2019.

Neto ED da S, Júnior ASM, Silva THCM, Ribeiro CBL., Nogueira J, Moreira L. Evaluation of oral ketorolac trometamol for pain control during intrauterine device insertion. Brazilian Journal of Development. 2001, 7(10), 95663–95672. <https://doi.org/10.34117/bjdv7n10-064a>

Njogu A, Qin S, Chen Y, Hu L, Luo Y. The effects of transcutaneous electrical nerve stimulation during the first stage of labor: a randomized controlled trial. BMC Pregnancy Childbirth. 2021 Feb 24;21(1):164. doi: 10.1186/s12884-021-03625-8. PMID: 33627077; PMCID: PMC7905652.

World Health Organization (WHO). (2000). Obesity: Preventing and Managing the Global Epidemic. Report of a WHO Consultation (WHO Technical Report Series 894). Geneva: World Health Organization.

Pedroso, Bruno & Gutierrez, Gustavo & Picinin, Claudia. (2016). WHOQOL-Pain: an instrument for assessing quality of life for people living with chronic physical pain. Brazilian Journal of Quality of Life. 8. 10.3895/rbqv.v8n3.4522.

Sikandar S, Dickenson AH. Visceral pain: the ins and outs, the ups and downs. Curr Opin Support Palliat Care. 2012 Mar;6(1):17-26. doi: 10.1097/SPC.0b013e32834f6ec9. PMID: 22246042; PMCID: PMC3272481.

Soares AM. Special Topics in Health Sciences: theory, methods and practices 6 [Internet]. 1st ^ed^ . AYA Editora; 2022 [cited May 31, 2024]. Available from: <https://ayaeditora.com.br/Livro/20605>

Tomazoni SS, Costa L da CM, Guimarães L de S, Araujo AC, Nascimento DP, Medeiros FC de, et al. Effects of photobiomodulation therapy in patients with chronic non-specific low back pain: protocol for a randomised placebo-controlled trial. BMJ Open. October 1, ^2017^ ;7(10):e017202.

Traverzim MADS, Makabe S, Silva DFT, Pavani C, Bussadori SK, Fernandes KSP, et al. Effect of led photobiomodulation on analgesia during labor: Study protocol for a randomized clinical trial. Medicine. Jun 2018;97(25):e11120.
