# Supplementary material for: Photobiomodulation for pain management during placement of the copper T 380 intrauterine device: Protocol for a randomized, double-blind controlled trial
Source: PLoS One. 2026 May 28;21(5):e0349031. doi: 10.1371/journal.pone.0349031 (PMC13218537; doi:10.1371/journal.pone.0349031)
Supplement: S4 File — This is the S3 File legend; there is no legend. (PDF) [file pone.0349031.s004.pdf]

PARECER CONSUBSTANCIADO DO CEP

DADOS DO PROJETO DE PESQUISA

Título da Pesquisa: EFEITO DA FOTOBIOMODULAÇÃO NA REDUÇÃO DA DOR DURANTE A INSERÇÃO DE DIU DE COBRE T 380 PARA A CONTRACEPÇÃO: ESTUDO CLÍNICO CONTROLADO RANDOMIZADO

Pesquisador: ANNA CAROLINA NUNES FERRAZ

Área Temática: Equipamentos e dispositivos terapêuticos, novos ou não registrados no País;

Versão: 1

CAAE: 85867925.5.0000.5551

Instituição Proponente: SAO PAULO SECRETARIA DA SAUDE

Patrocinador Principal: Financiamento Próprio

DADOS DO PARECER

Número do Parecer: 7.367.867

Apresentação do Projeto:

As informações elencadas nos campos "Apresentação do Projeto", "Objetivo da Pesquisa" e "Avaliação dos Riscos e Benefícios" foram extraídas do arquivo PB_INFORMAÇÕES_BÁSICAS_DO_PROJETO_2473758.pdf de 15/01/2025.

Resumo: A gravidez não planejada afeta até 65% das mulheres em algumas regiões do Brasil, elevando os riscos de abortos inseguros e contribuindo para a mortalidade materna. O DIU de cobre é uma alternativa contraceptiva eficaz e de longa duração, mas seu uso ainda é limitado no Brasil, abrangendo apenas 4,4% das mulheres em idade reprodutiva. Uma das principais barreiras é a dor associada à sua inserção, que gera medo e baixa adesão ao método. Como a dor pode ser de origem visceral ou somática, abordagens tradicionais como anti-inflamatórios e anestésicos mostram resultados inconclusivos na redução desse desconforto. A fotobiomodulação (FBM) promove efeitos anti-inflamatórios e analgésicos, apresentando resultados positivos no controle da dor pélvica em outros contextos clínicos, como o trabalho de parto. O objetivo deste estudo é avaliar a eficácia da FBM como método analgésico preemptivo na inserção do DIU de cobre T 380. Será realizado um ensaio clínico randomizado, duplo-cego, com 72 participantes distribuídas aleatoriamente entre grupo experimental (n=36) - FBM ativa e grupo controle (n=36) - simulação da FBM. As pacientes seguirão o protocolo de

inserção do DIU conforme diretrizes do Ministério da Saúde. A dor será avaliada em diferentes momentos utilizando a Escala Visual Analógica (EVA) durante as fases de inserção (Pozzi, Histereometria e a inserção) 5 e 15 minutos, 24 e 48 horas após a inserção do DIU. Além disso, serão investigados o uso de analgésicos e qualidade de vida (WHOQOL-100) no período de 48 horas, níveis de ansiedade (GAD-7), satisfação com o procedimento logo após a inserção (15 minutos) e efeitos adversos e colaterais em um período de 48 horas. Também será avaliada a duração da dor em horas desde o momento da inserção do DIU até o seu término. E o índice de sucesso do procedimento. A análise estatística será realizada no software SPSS versão 24.0, com nível de significância de 5% ($p < 0,05$). A normalidade dos dados será avaliada pelo teste de Shapiro-Wilk. Testes t de Student ou Mann-Whitney serão usados para variáveis contínuas, e o teste qui-quadrado ou exato de Fisher para variáveis categóricas. A dor será analisada pelo teste de Friedman e a regressão logística avaliará associações entre grupos e efeitos adversos. A análise estatística será realizada com um nível de significância de 5%. A normalidade dos dados será avaliada pelo teste de Shapiro-Wilk. Para a análise da dor (EVA) e de variáveis como ansiedade e qualidade de vida, será aplicado o teste de Friedman. O uso de analgésicos será avaliado por ANOVA para medidas repetidas. Os efeitos adversos serão analisados por regressão logística. O tempo para cessar o desconforto abdominal será estimado por meio da análise de Kaplan-Meier, e o sucesso de inserção do DIU será comparado utilizando o teste qui-quadrado.

Introdução:

A gestação não planejada acomete até 65% das mulheres (Costa et al., 2022) em algumas regiões do país, apesar das políticas públicas voltadas para a garantia do direito reprodutivo. A gravidez não planejada pode levar à realização de aborto em condições inseguras e à má assistência durante o pré-natal, que são causas importantes de mortalidade materna, por isso, fazem-se necessárias as ações de planejamento reprodutivo e garantia de acesso aos diversos métodos contraceptivos (Costa et al., 2022). Os métodos contraceptivos reversíveis de ação prolongada, como o dispositivo intrauterino (DIU) de cobre ajuda a reduzir o risco de gravidez indesejada em longo prazo, especialmente entre mulheres com menor escolaridade e baixo nível socioeconômico (Neto et al., 2021).Segundo a Pesquisa Nacional de Saúde de 2019, entre as mulheres de 15 a 49 anos que ainda menstruavam e que tinham sido sexualmente ativas nos últimos 12 meses 40,6% usavam pílula anticoncepcional, 22,9% usavam algum método de esterilização (17,3% a laqueadura e 5,6% a vasectomia) e apenas 4,4% usavam DIU apesar de

todos os seus aspectos positivos em relação aos outros métodos (Manual Técnico para Profissionais de Saúde,2018). Entre as indicações para o DIU podemos citar o desejo de método anticoncepcional de longa duração para mulheres em idade reprodutiva, incluindo a adolescência. É um método anticoncepcional de emergência, podendo ser colocado a qualquer momento do ciclo, controla o sangramento uterino aumentado (DIU hormonal), controla a dismenorreia (DIU hormonal), é uma opção contraceptiva para mulheres com histórico pessoal e familiar de trombose, mantém a anticoncepção pré captação de óvulos (reprodução assistida), sem piorar o desfecho do procedimento (Ministério da Saúde, 2018). Podemos citar como contraindicações do DIU, a distorção importante da cavidade uterina, doença inflamatória pélvica ativa, gravidez conhecida ou suspeita, doença de Wilson ou alergia ao cobre e sangramento uterino anormal sem causa definida (Ministério da Saúde, 2018). Para inserção de um dispositivo intrauterino é necessário que o profissional de saúde saiba que a mulher não está grávida e que ela não apresente nenhum sinal ou sintoma de gravidez. São utilizados alguns critérios para essa definição: A inserção pode ser feita durante a menstruação ou até 7 dias após o início da menstruação normal; não ter tido relações sexuais desde o início da última menstruação normal; ter usado corretamente e consistentemente um método confiável de contracepção; estar a menos de 7 dias após um abordo espontâneo ou induzido; dentro de 4 semanas no pós-parto; ou estar em aleitamento materno exclusivo ou quase exclusivo, em amenorreia e antes de 6 meses pós-parto (Ministério da Saúde, 2018).

Inserção do DIUO DIU de cobre é um método contraceptivo que deveria ser amplamente ofertado e inserido nas Unidades Básicas de Saúde (UBS) e Ambulatórios Médicos de Especialidades (AME). No entanto, no Brasil, há escassez de estudos que documentem e analisem a implementação desse método nos serviços de saúde. Diante do receio da inserção e utilização do DIU (Coleman et al., 2024), tanto por parte das mulheres quanto dos profissionais de saúde, é fundamental promover pesquisas que aprofundem o conhecimento sobre os procedimentos de inserção deste dispositivo nos serviços de saúde. Estes estudos podem contribuir para desmistificar o método garantindo maior adesão a essa forma de contracepção, que é eficaz e de baixo custo. (Almeida T, et al., 2023; Barreto D. et al,. 2020). Isto permitiria ampliar sua oferta, garantindo maior acesso à população de baixa renda (Almeida T, et al., 2023 ; Barreto D. et al,. 2020). Uma das grandes barreiras para a inserção de DIU, é a dor durante a inserção e consequente medo da paciente de passar por esse procedimento (Almeida et al., 2023; Lopes, et al., 2015).

Inervação/ Medula espinhal

A medula espinhal, é responsável pela inervação sensitiva de pele, músculos, articulações e vísceras e cada um desses grupos é chamado respectivamente de dermátomo, miótomo, esclerótomo ou viscerótomo. A dor no momento da inserção do DIU é de dois tipos: visceral e somática. A dor visceral é produzida pela dilatação do colo do útero, pois os nervos aferentes estão localizados entre as fibras dos músculos do colo do útero e os impulsos nervosos são transmitidos para a coluna espinhal por nervos sensoriais que são acompanhados por nervos simpáticos, sendo os dermatomos de T10, T11, T12 e L1 (plexo hipogástrico superior) diretamente envolvidos na percepção da dor (Erdo-an et al., 2023).A dor somática resulta da distensão da musculatura do assoalho pélvico, vagina e períneo, e os impulsos dolorosos são conduzidas pelos nervos pudendos, sendo os dermátomos S2, S3, S4, os mais importantes na percepção da dor (Erdo-an et al., 2023).A neuroanatomia mostra que os nervos da vagina e o útero são derivados do plexo nervoso uterovaginal, que é um dos plexos pélvicos que se estendem do plexo hipogástrico inferior até as vísceras pélvicas. Fibras aferentes simpáticas, parassimpáticas e viscerais atravessam esse plexo. (Moore,2014)A inervação simpática origina-se nos segmentos torácicos inferiores (T10) da medula espinal e atravessa os nervos esplâncnicos lombares e a série de plexos Inter mesentérico-hipogástrico-pélvicos. A inervação parassimpática origina-se nos segmentos S2-S4 da medula espinal e atravessa os nervos esplâncnicos pélvicos até o plexo hipogástrico inferior-uterovaginal. As fibras aferentes viscerais que conduzem impulsos de dor do fundo e do corpo do útero (acima da linha de dor pélvica) intraperitoneais seguem a inervação simpática retrógrada para chegar aos corpos celulares nos gânglios sensitivos de nervos espinais torácicos inferiores-lombares superiores. As fibras aferentes que conduzem impulsos de dor do colo do útero e da vagina (abaixo da linha de dor pélvica) subperitoneais seguem as fibras parassimpáticas retrogradamente através dos plexos uterovaginal e hipogástrico inferior e dos nervos esplâncnicos pélvicos para chegar aos corpos celulares nos gânglios sensitivos dos nervos espinais S2-S4. As duas diferentes vias seguidas por fibras de dor visceral são clinicamente importantes porque propiciam vários tipos de anestesia durante o parto, procedimentos pélvicos e controle de dores crônicas na região pélvica. Todas as fibras aferentes viscerais do útero e da vagina não relacionadas com a dor (aquelas que conduzem sensações inconscientes) também seguem a última via. (Moore, 2014) Métodos analgésicos Já foram realizados diversos estudos para tentar controlar e diminuir o desconforto durante a inserção do DIU (Lopes et al., 2015; Neto ED da S et al, 2021; Almeida T. et al, 2023; Erdo-an P et al, 2023), mas sempre com resultados inconclusivos, seja com uso de anti-inflamatórios não hormonais (AINES) (Lopes et al., 2015;

Neto ED da S et al, 2021; Almeida T. et al, 2023; Erdo-an P et al, 2023), uso de anestésicos locais (Lopes et al., 2015), ou preparo de colo (Lopes et al., 2015; Neto ED da S et al, 2021; Almeida T. et al, 2023; Erdo-an P et al, 2023).Em revisão da Cohrane (Lopes et al., 2015), foi demonstrado que o gel de lidocaína a 2%, o misoprostol e a maioria dos AINEs (antiinflamatórios não esteroides) não ajudaram a reduzir a dor e que essas intervenções são ineficazes, não necessitando novas pesquisas sobre o assunto. Algumas formulações de lidocaína, tramadol e naproxeno apresentaram algum efeito na redução da dor relacionada à inserção do DIU em alguns grupos específicos. A maior parte das evidências de eficácia foi de qualidade moderada, proveniente de ensaios isolados. Com a falta de evidência cientifica sobre a eficácia da analgesia local para diminuir a dor na inserção de DIU, procuramos outras formas de analgesia para controle de dor pélvica aguda, crônica e trabalho de parto. Foram encontrados trabalhos que mostram que o bloqueio paravertebral entre T10 e S4 com anestésicos, água destilada e fotobiomodulação apresentam algum nível de resultados. (Traverzim et al., 2018; Almeida et al., 2023; Neto et al., 2021). Alguns métodos de estimulação paravertebral têm sido eficazes no controle de dores pélvicas, especialmente durante o trabalho de parto. Entre eles podemos citar a Estimulação Elétrica Nervosa Transcutânea (TENS): A TENS tem sido amplamente utilizada para analgesia no parto. Embora o mecanismo exato ainda não seja completamente compreendido (Njogu et al., 2021), estudos comprovam sua eficácia e segurança. A aplicação da TENS de alta frequência (80 a 100 Hz) e largura de pulso de 350 microssegundos, com dois pares de eletrodos posicionados entre os níveis paravertebrais T10-L1 e S2-S4 durante a fase ativa do trabalho de parto, resultou em redução significativa da dor, conforme avaliado pela Escala Analógica Visual (VAS) (Soares et al., 2022). Como método anestésico também temos o bloqueio anestésico. O bloqueio anestésico paraespinhoso consiste em anestesiar o segmento espinhal entre T10 e L2, responsáveis pela inervação das vísceras pélvicas. Esse método proporciona alívio rápido para dores pélvicas agudas, mas não é indicado para o manejo da dor crônica (Rosa et al., 2013).Em um estudo realizado em 2018 no Conjunto Hospitalar do Mandaqui, em colaboração com o serviço de Biofotônica da Uninove, a irradiação com LED vermelho e infravermelho na região paravertebral entre T10 e S4 demonstrou ser eficaz na analgesia durante o trabalho de parto, promovendo conforto e redução da dor pélvica (Traverzim et al., 2018).Escolha do comparador A manipulação do colo e passagem do dispositivo pelo orifício interno pode ser desconfortável para algumas mulheres. O artigo de Tabatabaei et al. (2024) discute a dor associada à inserção de Dispositivos Intrauterinos (DIUs) como um fator que deve ser considerado cuidadosamente

para evitar complicações. Dores leves a moderadas são consideradas normais durante a inserção. Entretanto dores severas ou persistentes são indicativas de complicações como perfuração uterina ou dano visceral. Profissionais de saúde não devem subestimar a dor relatada pelas pacientes ou não realizar os exames complementares necessários para verificar a correta posição do DIU, o que pode resultar em diagnósticos tardios e reinserções desnecessárias (Tabatabaei et al. 2024). Anestesia mais profundas são contraindicadas, pois um pequeno nível de dor é desejável, como descrito acima. Algumas estratégias podem ser utilizadas, apesar de não terem eficácia científica comprovada, como a administração de antiinflamatório não esteróide (AINE) por via oral, antes da inserção do DIU. O grupo controle em trabalhos de inserção de DIU pode ser com placebo, ausência de intervenção ou outra intervenção ativa (Lopes, 2015). Fotobiomodulação no controle da dor Estudos recentes têm demonstrado a eficácia da fotobiomodulação no tratamento de dores lombares, utilizando diferentes comprimentos de onda. Lin et al. (2020) observaram bons resultados com a irradiação da região toracolombar, utilizando comprimentos de onda entre 630 e 850 nm. Em outro estudo, Tomazoni et al. (2020) empregaram 490 nm com correção para 570 nm e constataram alterações na microcirculação e no controle da liberação de interleucinas inflamatórias. Adicionalmente, a aplicação de 905 nm durante 3 minutos, cobrindo uma área maior (T11 a S1), foi eficaz para o manejo da dor lombar crônica (Tomazoni et al., 2017). Em outro estudo, Traverzim (2018) utilizou irradiação entre T10 e S4 para analgesia no trabalho de parto, aplicando energia de 1,5 J em comprimentos de onda vermelho e infravermelho, com resultados promissores. Dado que resultados promissores têm sido observados com o uso de terapias não medicamentosas para inserção de DIU (Gemzell-Danielsson, 2019) para analgesia durante o parto (Traverzim, 2018), e considerando que o Ministério da Saúde recomenda a inserção do DIU intrauterino sem o uso de qualquer recurso analgésico, torna-se essencial investigar formas de minimizar a dor relatada pelas mulheres durante e após a inserção do dispositivo. Estudos adicionais são necessários para explorar alternativas que possam reduzir esse desconforto, incentivando mais mulheres a aderirem a esse método contraceptivo, que é eficaz e de baixo custo, promovendo um impacto positivo na saúde pública brasileira.

Hipótese: Hipótese experimental O uso da FBM é capaz de diminuir a percepção da dor durante a inserção de DIU de cobre T 380 para contracepção.

Metodologia

Trata-se de um ensaio clínico único-centro randomizado, duplo-cego controlado com dois grupos paralelos, de superioridade delineado conforme o os critérios SPIRIT Statement (https:

//www.spirit-statement.org/) O projeto será submetido ao Comitê de Ética em Pesquisa (CEP) do Conjunto Hospitalar do Mandaqui da cidade de São Paulo. Qualquer intercorrência ou alteração durante o estudo será reportada e esclarecida ao CEP e nas futuras publicações deste estudo. Após explicação verbal pela pesquisadora principal, e por escrito do estudo, as participantes que aceitarem participar, assinarão o Termo de Consentimento Livre e Esclarecido (TCLE). As participantes que desejarem receber os dados da pesquisa, informarão o seu e-mail no TCLE e o artigo completo será fornecido assim que publicado. Os tratamentos serão realizados no Conjunto Hospitalar do Mandaqui, situado na Zona Norte da cidade de São Paulo, Brasil, no período de novembro 2024 a abril de 2026 pela médica Ginecologista com experiência de mais de 10 anos. O projeto será registrado na Plataforma Clinicaltrials (https://clinicaltrials.gov/).

Calibração/treinamento

Haverá apenas uma examinadora que avaliará 5 mulheres, as quais não farão parte do estudo. Serão feitas avaliações clínicas de dor exatamente como proposto pelo presente trabalho. A pesquisadora principal é médica ginecologista com experiencia de mais de 10 anos na área e fará todas as inserções de DIU e as avaliações pós-operatórias. Neste trabalho, a pesquisadora principal será treinada para avaliar os seguintes desfechos: ansiedade, por meio do questionário Generalized Anxiety Disorder 7 (GAD-7); qualidade de vida, utilizando o instrumento WHOQOL-Pain; e a satisfação das pacientes, com base em um questionário estruturado, conforme o estudo de Lopes (2015).

Cálculo do tamanho da amostra

O tamanho total da amostra será de 60 pacientes por grupo. Esse valor foi calculado para fornecer um poder de 95% (- = 0,05) e um tamanho de efeito de 0.6421598. Para determinar o número de pacientes em cada grupo, foi realizado um cálculo amostral com base na variabilidade dos resultados de 1 artigo que avaliou o desfecho dor, medida em milímetros (mm) com a Escala analógica visual. Foi considerado o mesmo intervalo de tempo do estudo (24h). Em um grupo foi utilizada a FBM e obteve-se média de dor em milímetros de 1.91±1.76 e o outro grupo não foi utilizada a FBM e obteve-se média de dor de 3.14±2.04. Situação idêntica ao desfecho primário utilizado neste estudo. Utilizando o método de teste t-two tails, a amostra necessária será 60 indivíduos, sendo 30 por grupo. . Descrição da amostra Serão selecionadas mulheres em idade reprodutiva, encaminhadas ao hospital para contracepção, das UBSs da Zona Norte de São Paulo ou do Pronto Socorro ginecológico do Conjunto Hospitalar do Mandaqui.

Critério de Inclusão:

Endereço: Rua Voluntários da Pátria, 4301 - Prédio 03 - Casa Azul
Bairro: SANTANA **CEP:** 02.401-400
UF: SP **Município:** SAO PAULO
Telefone: (11)2281-5147 **Fax:** (11)2281-5179 **E-mail:** chm-cep@saude.sp.gov.br

-Participantes de 18-50 anos,-Sexo feminino,
- Sem predileção de raça ou nível socioeconômico,
- Nulíparas ou multíparas

Critério de Exclusão:
-Gravidez conhecida ou suspeita,
-Dor crônica diagnosticada,
-Infecção ativa local,
-Qualquer medicamento para dor nas últimas 12 horas,
-Contraindicação conhecida para a colocação do DIU (distorção importante da cavidade uterina, doença inflamatória pélvica ativa, doença de Wilson),
-Alergia ao cobre (Ministério da Saúde, 2018),
-Sangramento uterino anormal sem causa definida (Ministério da Saúde, 2018),
-Com qualquer alteração na região lombar como por exemplo: neoplasias ativas, osteomielite instalada, qualquer lesão tissular profunda preexistente, com necrose ou infectada,
-- Com histórico de fotossensibilidade.
-Recusa do participante

Metodologia de Análise de Dados:
A análise estatística será realizada utilizando o software Statistical Package for the Social Sciences (SPSS) versão 24.0 ou software equivalente. O nível de significância será estabelecido em 5% ($p < 0,05$) para todos os testes. A normalidade dos dados será avaliada pelo teste de Shapiro-Wilk. Variáveis contínuas serão descritas por meio de média e desvio-padrão, ou mediana e intervalo interquartílico, conforme a distribuição dos dados. Variáveis categóricas serão apresentadas como frequências absolutas e relativas. Para a comparação entre os grupos experimental (FBM ativa) e controle (simulação de FBM) será utilizado o teste de Mann-Whitney para variáveis contínuas com distribuição não normal. Variáveis categóricas serão comparadas utilizando o teste qui-quadrado ou teste exato de Fisher, conforme valor mínimo esperado seja menor que 5. A análise das variáveis relacionadas à dor (intensidade medida pela Escala Visual Analógica ¿ EVA) em diferentes momentos (baseline, durante a inserção, 5 e 15 minutos, 24 e 48 horas após o procedimento) será realizada por meio do teste de Friedman ou por modelo linear generalizado, ajustando-se para possíveis covariáveis. Para avaliar a ansiedade (GAD-7), qualidade de vida (WHOQOL-100) e satisfação com o procedimento, será

Endereço: Rua Voluntários da Pátria, 4301 - Prédio 03 - Casa Azul
Bairro: SANTANA **CEP:** 02.401-400
UF: SP **Município:** SAO PAULO
Telefone: (11)2281-5147 **Fax:** (11)2281-5179 **E-mail:** chm-cep@saude.sp.gov.br

realizado o teste Mann-Whitney para comparação entre os grupos nos diferentes momentos de avaliação. O uso de analgésicos pós-procedimento será comparado entre os grupos por meio de análise de variância (ANOVA) para medidas repetidas, considerando o número de comprimidos ingeridos em cada momento (24h e 48h). Adicionalmente, para verificar a associação entre os grupos e a ocorrência de efeitos adversos e colaterais, será realizada regressão logística, considerando o grupo como variável preditora e os efeitos adversos como variável resposta. Por fim, será realizada uma análise de Kaplan-Meier para estimar o tempo necessário para cessar o desconforto abdominal (cólica) e o sucesso de inserção do DIU será analisado por meio de comparação de proporções entre os grupos utilizando teste qui-quadrado.
Desfecho Primário: Melhora do desconforto ocasionado pela inserção do DIU com método de fácil utilização em qualquer contexto de saúde.

Objetivo da Pesquisa:

Objetivo Primário:
- Avaliação da dor (EVA) durante as fases de inserção do DIU (Pozzi, Histereometria e inserção do DIU) 5 e 15 minutos, 24 e 48 horas após a inserção do DIU.
- Quantidade de medicamentos (paracetamol) ingeridos pela paciente no período de 48 horas.
- Avaliação da ansiedade das pacientes por meio do instrumento Generalized Anxiety Disorder 7 (GAD-7)
- Avaliação da qualidade de vida das pacientes por meio do instrumento WHOQOL-Pain
- Satisfação das pacientes por meio de questionário estruturado (Lopes, 2015)
- Tempo para cessar o desconforto abdominal
- Efeitos adversos (ex: sangramento, desmaio, alergia)
- Efeitos colaterais (cólicas, calafrio, anestesia da língua)
- Falha na inserção do DIU

Avaliação dos Riscos e Benefícios:

Riscos:

- Efeitos adversos (Perfuração Uterina, Deslocamento do DIU, Dor Abdominal e Sangramento Vaginal aumentado, alergia). Será realizada uma pergunta aberta para que a paciente responda abertamente sobre os efeitos adversos e depois serão elencados nominalmente para que ela possa se lembrar de algum efeito que porventura tenha esquecido de relatar.

- Efeitos colaterais (cólicas, dor leve, sangramento leve, anestesia da língua). Será realizada uma pergunta aberta para que a paciente responda abertamente sobre os efeitos colaterais e depois serão elencados nominalmente para que ela possa se lembrar de algum efeito que porventura tenha esquecido de relatar. Benefícios: reduzir o desconforto associado à inserção do DIU, promovendo maior aceitação e adesão ao método. A fotobiomodulação (FBM), por sua comprovada ação anti-inflamatória e analgésica, surge como uma alternativa promissora. Ao irradiar as terminações nervosas da região toracolombar, a FBM pode inibir a propagação da dor para órgãos somáticos e viscerais, proporcionando um procedimento mais confortável e uma recuperação mais rápida. Assim, este estudo justifica-se pela necessidade de avaliar se a utilização da FBM durante a inserção do DIU-Cu T 380 em sessão única pode contribuir para a redução da dor intra e pós-procedimento, promovendo maior conforto e bem-estar às mulheres e, consequentemente, encorajando a adoção de um método contraceptivo seguro e de baixo custo, com impacto positivo na saúde pública brasileira.

Comentários e Considerações sobre a Pesquisa:

Projeto original PO - Versão 1- Ensaio clinico randomizado duplo-cego.

A fotobiomodulação é considerada como um procedimetno seguro amplamente embasado em literatura. Os eventuais riscos relacionados ao protocolo da pesquisa foram adequadamente descritos. Os criterios de inclusão e exclusão foram devidamente apresentados.

Não se verificaram pendências éticas no delineamento desta pesquisa.

Considerações sobre os Termos de apresentação obrigatória:

O TCLE possui todas as exigências determinadas pela resolução CONEP no. 466/2012. Não se verificaram pendências nestes quesito.

Recomendações:

Incluir referências que demonstrem a segurança da aplicação de fotobiomodulação em mucosa vaginal.

Conclusões ou Pendências e Lista de Inadequações:

Não foram verificadas inadequações ou pendências e o relator propõe que esta pesquisa seja considerada APROVADA.

Uma vez finalizada a pesquisa, enviar ao CEP o Relatório Final sob forma de Notificação, contendo o resumo do trabalho, informando número de participantes, conclusões e dados do status da publicação (enviado, aceito ou publicado).

Considerações Finais a critério do CEP:

O Colegiado acolheu na íntegra o parecer emitido pelo membro relator.

O presente projeto, seguiu nesta data para análise da CONEP e só tem o seu início autorizado após a aprovação pela mesma.

Este parecer foi elaborado baseado nos documentos abaixo relacionados:

Tipo Documento	Arquivo	Postagem	Autor	Situação
Informações Básicas do Projeto	PB_INFORMAÇÕES_BÁSICAS_DO_PROJETO_2473758.pdf	15/01/2025 16:48:00		Aceito
Projeto Detalhado / Brochura Investigador	ProjetoFerrazACNrev.docx	15/01/2025 16:47:23	ANNA CAROLINA NUNES FERRAZ	Aceito
TCLE / Termos de Assentimento / Justificativa de Ausência	TCLErev.docx	15/01/2025 16:46:38	ANNA CAROLINA NUNES FERRAZ	Aceito
Folha de Rosto	Folhaderosto.pdf	15/01/2025 16:43:18	ANNA CAROLINA NUNES FERRAZ	Aceito

Situação do Parecer:

Aprovado

Necessita Apreciação da CONEP:

Sim

SAO PAULO, 07 de Fevereiro de 2025

Assinado por:

RENATO CARDOSO
(Coordenador(a))

Endereço: Rua Voluntários da Pátria, 4301 - Prédio 03 - Casa Azul
Bairro: SANTANA **CEP:** 02.401-400
UF: SP **Município:** SAO PAULO
Telefone: (11)2281-5147 **Fax:** (11)2281-5179 **E-mail:** chm-cep@saude.sp.gov.br
