# Supplementary material for: Photobiomodulation for pain management during placement of the copper T 380 intrauterine device: Protocol for a randomized, double-blind controlled trial
Source: PLoS One. 2026 May 28;21(5):e0349031. doi: 10.1371/journal.pone.0349031 (PMC13218537; doi:10.1371/journal.pone.0349031)
Supplement: S5 File — This is the S4 File legend; there is no legend. (PDF) [file pone.0349031.s005.pdf]

Machine Translated by Google

HOSPITAL COMPLEX OF MANDAQUI - CHM

CONSUBSTANTIATED OPINION OF THE CEP

RESEARCH PROJECT DATA

Search Title: EFFECT OF PHOTOBIOMODULATION ON PAIN REDUCTION DURING INSERTION OF COPPER IUD T 380 FOR CONTRACEPTION: A STUDY RANDOMIZED CONTROLLED CLINICAL TRIAL

Researcher: ANNA CAROLINA NUNES FERRAZ

Thematic Area: Therapeutic equipment and devices, new or not registered in the Country;

Version: 1

CAAE: 85867925.5.0000.5551

Proposing Institution: SAO PAULO DEPARTMENT OF HEALTH

Main Sponsor: Own Financing

OPINION DATA

Opinion Number: 7,367,867

Project Presentation: The

information listed in the fields "Project Presentation", "Research Objective" and "Risk and Benefit Assessment" were extracted from the file PB_INFORMAÇÕES_BÁSICAS_DO_PROJETO_2473758.pdf from 01/15/2025.

Abstract: Unplanned pregnancy affects up to 65% of women in some regions of Brazil, increasing the risk of unsafe abortions and contributing to maternal mortality. The copper IUD is an effective and long-acting contraceptive alternative, but its use is still limited in Brazil, reaching only 4.4% of women of reproductive age. One of the main barriers is the pain associated with its insertion, which generates fear and low adherence to the method. Since pain can be of visceral or somatic origin, traditional approaches such as anti-inflammatories and anesthetics show inconclusive results in reducing this discomfort. Photobiomodulation (PBM) promotes anti-inflammatory and analgesic effects, presenting positive results in the control of pelvic pain in other clinical contexts, such as labor.

The aim of this study is to evaluate the efficacy of FBM as a preemptive analgesic method in the insertion of the T 380 copper IUD. A randomized, double-blind clinical trial will be conducted with 72 participants randomly distributed between the experimental group (n=36) - active FBM and the control group (n=36) - FBM simulation. Patients will follow the protocol of

Machine Translated by Google

IUD insertion according to the guidelines of the Ministry of Health. Pain will be assessed at different times using the Visual Analog Scale (VAS) during the insertion phases (Pozzi, Hystereometry and insertion) 5 and 15 minutes, 24 and 48 hours after IUD insertion. In addition, the use of analgesics and quality of life (WHOQOL-100) in the 48-hour period, anxiety levels (GAD-7), satisfaction with the procedure immediately after insertion (15 minutes) and adverse and side effects in a 48-hour period will be investigated.
The duration of pain in hours from the moment the IUD is inserted until it is finished will also be assessed.
And the success rate of the procedure. Statistical analysis will be performed using SPSS software version 24.0, with a significance level of 5% ($p < 0.05$). Data normality will be assessed by the Shapiro-Wilk test. Student's t-test or Mann-Whitney test will be used for continuous variables, and the chi-square or Fisher's exact test for categorical variables. Pain will be analyzed by the Friedman test, and logistic regression will assess associations between groups and adverse effects. Statistical analysis will be performed with a significance level of 5%. Data normality will be assessed by the Shapiro-Wilk test. The Friedman test will be applied to analyze pain (VAS) and variables such as anxiety and quality of life.
Analgesic use will be assessed by repeated-measures ANOVA. Adverse effects will be analyzed by logistic regression. Time to cessation of abdominal discomfort will be estimated by Kaplan-Meier analysis, and IUD insertion success will be compared using the chi-square test.

Introduction:
Unplanned pregnancy affects up to 65% of women (Costa et al., 2022) in some regions of the country, despite public policies aimed at guaranteeing reproductive rights. Unplanned pregnancy can lead to abortion in unsafe conditions and poor care during prenatal care, which are important causes of maternal mortality. Therefore, reproductive planning actions and guaranteed access to various contraceptive methods are necessary (Costa et al., 2022). Long-acting reversible contraceptive methods, such as the copper intrauterine device (IUD), help reduce the risk of unwanted pregnancy in the long term, especially among women with less education and low socioeconomic status (Neto et al., 2021). According to the 2019 National Health Survey, among women aged 15 to 49 who were still menstruating and who had been sexually active in the last 12 months, 40.6% used contraceptive pills, 22.9% used some sterilization method (17.3% tubal ligation and 5.6% vasectomy) and only 4.4% used IUDs despite

all its positive aspects in relation to other methods (Technical Manual for Health Professionals, 2018). Among the indications for the IUD we can mention the desire for a long-term contraceptive method for women of reproductive age, including adolescence. It is an emergency contraceptive method, and can be inserted at any time of the cycle, controls increased uterine bleeding (hormonal IUD), controls dysmenorrhea (hormonal IUD), is a contraceptive option for women with a personal and family history of thrombosis, maintains contraception before egg retrieval (assisted reproduction), without worsening the outcome of the procedure (Ministry of Health, 2018). We can mention as contraindications of the IUD, significant distortion of the uterine cavity, pelvic inflammatory disease
active pregnancy, known or suspected pregnancy, Wilson's disease or copper allergy, and abnormal uterine bleeding with no defined cause (Ministry of Health, 2018). To insert an intrauterine device, the health professional must know that the woman is not pregnant and that she does not present any signs or symptoms of pregnancy. Some criteria are used for this definition: The insertion can be done during menstruation or up to 7 days after the start of normal menstruation; not having had sexual intercourse since the start of the last normal menstruation; having used a reliable method of contraception correctly and consistently; being less than 7 days after a spontaneous or induced abortion; within 4 weeks postpartum; or being exclusively or almost exclusively breastfed, in amenorrhea and before 6 months postpartum (Ministry of Health, 2018).

IUD InsertionThe copper IUD is a contraceptive method that should be widely offered and inserted in Basic Health Units (UBS) and Specialty Medical Outpatient Clinics (AME). However, in Brazil, there is a shortage of studies that document and analyze the implementation of this method in health services. Given the fear of inserting and using the IUD (Coleman et al., 2024), both on the part of women and health professionals, it is essential to promote research that deepens knowledge about the procedures for inserting this device in health services. These studies can contribute to demystifying the method, ensuring greater adherence to this form of contraception, which is effective and low-cost (Almeida T, et al., 2023; Barreto D. et al,. 2020). This would allow expanding its supply, ensuring greater access to the low-income population (Almeida T, et al., 2023 ; Barreto D. et al,. 2020). One of the major barriers to IUD insertion is the pain during insertion and the patient's consequent fear of undergoing this procedure (Almeida et al., 2023; Lopes, et al., 2015).

Innervation/ Spinal cord

The spinal cord is responsible for the sensory innervation of the skin, muscles, joints and viscera and each one of these groups is called respectively dermatome, myotome, sclerotome or viscerotome. Pain at the time of IUD insertion is of two types: visceral and somatic. Visceral pain is produced by dilation of the cervix, as the afferent nerves are located between the fibers of the cervical muscles and the nerve impulses are transmitted to the spinal column by sensory nerves that are accompanied by sympathetic nerves, with the dermatomes of T10, T11, T12 and L1 (superior hypogastric plexus) being directly involved in the perception of pain (Erdo-an et al., 2023). Somatic pain results from the distension of the muscles of the pelvic floor, vagina and perineum, and the painful impulses are conducted by the pudendal nerves, with the dermatomes S2, S3, S4 being the most important in the perception of pain (Erdo-an et al., 2023). Neuroanatomy shows that the nerves of the vagina and uterus are derived from the uterovaginal nerve plexus, which is one of the pelvic plexuses that extend from the hypogastric plexus. inferior to the pelvic viscera. Sympathetic, parasympathetic and visceral afferent fibers cross this plexus. (Moore, 2014) Sympathetic innervation originates in the lower thoracic segments (T10) of the spinal cord and crosses the lumbar splanchnic nerves and the series of intermesenteric-hypogastric-pelvic plexuses. Parasympathetic innervation originates in the S2-S4 segments of the spinal cord and crosses the pelvic splanchnic nerves to the inferior hypogastric-uterovaginal plexus. The visceral afferent fibers that carry pain impulses from the fundus and body of the uterus (above the pelvic pain line) intraperitoneally follow retrograde sympathetic innervation to reach cell bodies in the sensory ganglia of the lower thoracic-upper lumbar spinal nerves. The afferent fibers that carry pain impulses from the cervix and vagina (below the pelvic pain line) subperitoneally follow the parasympathetic fibers retrogradely through the uterovaginal and inferior hypogastric plexuses and the pelvic splanchnic nerves to reach cell bodies in the sensory ganglia of the S2-S4 spinal nerves. The two distinct pathways followed by visceral pain fibers are clinically important because they provide various types of anesthesia during childbirth, pelvic procedures, and management of chronic pelvic pain. All visceral afferent fibers from the uterus and vagina not related to pain (those that conduct unconscious sensations) also follow the latter pathway. (Moore, 2014) Analgesic methods Several studies have been carried out to try to control and reduce discomfort during IUD insertion (Lopes et al., 2015; Neto ED da S et al, 2021; Almeida T. et al, 2023; Erdo-an P et al, 2023), but always with inconclusive results, either with the use of non-hormonal anti-inflammatory drugs (NSAIDs) (Lopes et al., 2015;

Machine Translated by Google

Neto ED da S et al, 2021; Almeida T. et al, 2023; Erdo-an P et al, 2023), use of local anesthetics (Lopes et al., 2015), or cervix preparation (Lopes et al., 2015; Neto ED da S et al, 2021; Almeida T. et al, 2023; Erdo-an P et al, 2023). In a Cohrane review (Lopes et al., 2015), it was shown that 2% lidocaine gel, misoprostol, and most NSAIDs (non-steroidal anti-inflammatory drugs) did not help to reduce pain and that these interventions are ineffective, not requiring further research on the subject. Some formulations of lidocaine, tramadol, and naproxen showed some effect in reducing pain related to IUD insertion in some specific groups. Most of the evidence of efficacy was of moderate quality, coming from isolated trials. Given the lack of scientific evidence on the efficacy of local analgesia to reduce pain during IUD insertion, we looked for other forms of analgesia to control acute, chronic pelvic pain and labor. Studies were found showing that paravertebral blockade between T10 and S4 with anesthetics, distilled water and photobiomodulation present some level of results (Traverzim et al., 2018; Almeida et al., 2023; Neto et al., 2021). Some methods of paravertebral stimulation have been effective in controlling pelvic pain, especially during labor. Among them we can mention Transcutaneous Electrical Nerve Stimulation (TENS): TENS has been widely used for analgesia during labor. Although the exact mechanism is not yet fully understood (Njogu et al., 2021), studies prove its efficacy and safety. The application of high-frequency TENS (80 to 100 Hz) and a pulse width of 350 microseconds, with two pairs of electrodes positioned between the paravertebral levels T10-L1 and S2-S4 during the active phase of labor, resulted in a significant reduction in pain, as assessed by the Visual Analog Scale (VAS) (Soares et al., 2022). As an anesthetic method, we also have the anesthetic block. The paraspinal anesthetic block consists of anesthetizing the spinal segment between T10 and L2, responsible for the innervation of the pelvic viscera. This method provides rapid relief for acute pelvic pain, but is not indicated for the management of chronic pain (Rosa et al., 2013). In a study carried out in 2018 at the Mandaqui Hospital Complex, in collaboration with the Uninove Biophotonics service, irradiation with red and infrared LED in the paravertebral region between T10 and S4 proved to be effective in analgesia during labor, promoting comfort and reducing pelvic pain (Traverzim et al., 2018). Choice of comparator Manipulation of the cervix and passage of the device through the internal os can be uncomfortable for some women. The article by Tabatabaei et al. (2024) discusses the pain associated with the insertion of Intrauterine Devices (IUDs) as a factor that should be carefully considered

Machine Translated by Google

to avoid complications. Mild to moderate pain is considered normal during insertion.
However, severe or persistent pain is indicative of complications such as uterine perforation or visceral damage. Healthcare professionals should not underestimate the pain reported by patients or fail to perform the necessary additional tests to verify the correct position of the IUD, which may result in late diagnoses and unnecessary reinsertions (Tabatabaei et al. 2024). Deeper anesthesia is contraindicated, as a low level of pain is desirable, as described above. Some strategies can be used, although they have no proven scientific efficacy, such as the administration of oral nonsteroidal anti-inflammatory drugs (NSAIDs) before IUD insertion. The control group in studies of IUD insertion may be with placebo, no intervention or another active intervention (Lopes, 2015).
Photobiomodulation in pain control Recent studies have demonstrated the efficacy of photobiomodulation in the treatment of low back pain, using different wavelengths. Lin et al. (2020) observed good results with irradiation of the thoracolumbar region, using wavelengths between 630 and 850 nm. In another study, Tomazoni et al. (2020) used 490 nm with correction for 570 nm and found changes in microcirculation and in the control of the release of inflammatory interleukins. Additionally, the application of 905 nm for 3 minutes, covering a larger area (T11 to S1), was effective for the management of chronic low back pain (Tomazoni et al., 2017). In another study, Traverzim (2018) used irradiation between T10 and S4 for analgesia in labor, applying energy of 1.5 J in red and infrared wavelengths, with promising results. Given that promising results have been observed with the use of non-drug therapies for IUD insertion (Gemzell-Danielsson, 2019) for analgesia during labor (Traverzim, 2018), and considering that the Ministry of Health recommends the insertion of the intrauterine IUD without the use of any analgesic resource, it is essential to investigate ways to minimize the pain reported by women during and after insertion of the device. Additional studies are needed to explore alternatives that can reduce this discomfort, encouraging more women to adhere to this contraceptive method, which is effective and low-cost, promoting a positive impact on Brazilian public health.

Hypothesis: Experimental hypothesis The use of FBM is capable of reducing the perception of pain during insertion of copper IUD T 380 for contraception.
Methodology
This is a single-center, randomized, double-blind, controlled clinical trial with two parallel groups, of superiority designed according to the SPIRIT Statement criteria (https:

//www.spirit-statement.org/) The project will be submitted to the Research Ethics Committee (CEP) of the Mandaqui Hospital Complex in the city of São Paulo. Any complications or changes during the study will be reported and clarified to the CEP and in future publications of this study. After verbal explanation by the main researcher, and in writing of the study, the participants who agree to participate will sign the Free and Informed Consent Form (FICF). Participants who wish to receive the research data will inform their email in the FICF and the full article will be provided as soon as it is published. The treatments will be carried out at the Mandaqui Hospital Complex, located in the North Zone of the city of São Paulo, Brazil, from November 2024 to April 2026 by a Gynecologist with over 10 years of experience. The project will be registered on the Clinicaltrials Platform (https://clinicaltrials.gov/).

Calibration/training There will
be only one examiner who will evaluate 5 women, who will not be part of the study. Clinical assessments of pain will be carried out exactly as proposed in this study. The main researcher is
A gynecologist with over 10 years of experience in the field, she will perform all IUD insertions and postoperative evaluations. In this study, the lead researcher will be trained to assess the following outcomes: anxiety, using the Generalized Anxiety Disorder 7 (GAD-7) questionnaire; quality of life, using the WHOQOL-Pain instrument; and patient satisfaction, based on a structured questionnaire, according to the study by Lopes (2015).

Sample size calculation

The total sample size will be 60 patients per group. This value was calculated to provide a power of 95% (- = 0.05) and an effect size of 0.6421598. To determine the number of patients in each group, a sample calculation was performed based on the variability of the results of 1 article that evaluated the outcome pain, measured in millimeters (mm) with the Visual Analog Scale. The same time interval of the study (24h) was considered. In one group, FBM was used and a mean pain in millimeters of 1.91 ± 1.76 was obtained, and in the other group, FBM was not used and a mean pain of 3.14 ± 2.04 was obtained.

Identical situation to the primary outcome used in this study. Using the two-tailed t-test method, the required sample will be 60 individuals, with 30 per group. . Sample description Women of reproductive age, referred to the hospital for contraception, from the UBSs in the North Zone of São Paulo or from the Gynecological Emergency Room of the Mandaqui Hospital Complex will be selected.

Inclusion Criteria:

Machine Translated by Google

-Participants aged 18-50,-Female,

- No predilection for race or socioeconomic status,

- Nulliparous or multiparous

Exclusion Criteria:

-Known or suspected pregnancy,

-Diagnosed chronic pain,

-Active local infection,

-Any pain medication in the last 12 hours,

-Known contraindication for IUD placement (significant distortion of the uterine cavity, active pelvic inflammatory disease, Wilson's disease),

-Copper allergy (Ministry of Health, 2018),

-Abnormal uterine bleeding without a defined cause (Ministry of Health, 2018),

-With any alteration in the lumbar region such as: active neoplasms, established osteomyelitis, any pre-existing deep tissue lesion, with necrosis or infection,

-- With a history of photosensitivity.

-Participant refusal

Data Analysis Methodology: Statistical
analysis will be performed using the Statistical Package for the Social Sciences (SPSS) version 24.0 or equivalent software. The significance level will be set at 5% ($p < 0.05$) for all tests. Data normality will be assessed by the Shapiro-Wilk test. Continuous variables will be described by mean and standard deviation, or median and interquartile range, according to the data distribution. Categorical variables will be presented as absolute and relative frequencies. The Mann-Whitney test will be used to compare the experimental (active FBM) and control (FBM simulation) groups for continuous variables with non-normal distribution. Categorical variables will be compared using the chi-square test or Fisher's exact test, depending on whether the minimum expected value is less than 5. The analysis of variables related to pain (intensity measured by the Visual Analog Scale - VAS) at different times (baseline, during insertion, 5 and 15 minutes, 24 and 48 hours after the procedure) will be performed using the Friedman test or a generalized linear model, adjusting for possible covariates. To assess anxiety (GAD-7), quality of life (WHOQOL-100) and satisfaction with the procedure,

Address: Volunteers of the Nation Street, 4301 - Building 03 - Blue House
Neighborhood: SANTANA **ZIP Code:** 02.401-400
State: SP **Municipality:** SAO PAULO
Telephone: (11)2281-5147 **Fax:** (11)2281-5179 **Email:** chm-cep@saude.sp.gov.br

Machine Translated by Google

The Mann-Whitney test was performed to compare the groups at different evaluation times. The use of post-procedure analgesics will be compared between the groups using analysis of variance (ANOVA) for repeated measures, considering the number of tablets ingested at each time point (24h and 48h). Additionally, to verify the association between the groups and the occurrence of adverse and side effects, logistic regression will be performed, considering the group as the predictor variable and the adverse effects as the response variable. Finally, a Kaplan-Meier analysis will be performed to estimate the time required to cease abdominal discomfort (colic) and the success of IUD insertion will be analyzed by comparing proportions between the groups using the chi-square test.
Primary Outcome: Improvement in discomfort caused by IUD insertion with an easy-to-use method in any health context.

Research Objective:
Primary Objective:
- Pain assessment (VAS) during the IUD insertion phases (Pozzi, Hystereometry and IUD insertion) 5 and 15 minutes, 24 and 48 hours after IUD insertion.
- Quantity of medication (paracetamol) taken by the patient in the 48-hour period.
- Assessment of patients' anxiety using the Generalized Anxiety Disorder 7 (GAD-7) instrument
- Assessment of patients' quality of life using the WHOQOL-Pain instrument - Patient satisfaction using a structured questionnaire (Lopes, 2015)
- Time to stop abdominal discomfort - Adverse effects (e.g. bleeding, fainting, allergy)
- Side effects (cramps, chills, tongue numbness)
- IUD insertion failure

Risk and Benefit Assessment:
Risks:
- Adverse effects (Uterine perforation, IUD displacement, abdominal pain and increased vaginal bleeding, allergy). An open question will be asked so that the patient can respond openly about the adverse effects and then they will be listed by name so that she can remember any effect that she may have forgotten to report.

Machine Translated by Google

- Side effects (cramps, mild pain, mild bleeding, numbness of the tongue). An open-ended question will be asked so that the patient can respond openly about the side effects and then they will be listed by name so that she can remember any effects that she may have forgotten to report.

Benefits: reducing discomfort associated with IUD insertion, promoting greater acceptance and adherence to the method. Photobiomodulation (PBM), due to its proven anti-inflammatory and analgesic action, appears as a promising alternative. By irradiating the nerve endings of the thoracolumbar region, PBM can inhibit the propagation of pain to somatic and visceral organs, providing a more comfortable procedure and faster recovery. Thus, this study is justified by the need to evaluate whether the use of PBM during the insertion of the Cu-IUD T 380 in a single session can contribute to the reduction of intra- and post-procedure pain, promoting greater comfort and well-being for women and, consequently, encouraging the adoption of a safe and low-cost contraceptive method, with a positive impact on public health.

Brazilian.

Comments and Considerations on the Research: Original PO

Project - Version 1 - Double-blind randomized clinical trial.

Photobiomodulation is considered a safe procedure, widely supported by the literature. Any risks related to the research protocol were adequately described. Inclusion and exclusion criteria were duly presented.

There were no ethical issues in the design of this research.

Considerations on the Mandatory Presentation Terms: The TCLE has all the requirements determined by CONEP resolution no. 466/2012. There were no pending issues in these matters.

Recommendations:

Include references that demonstrate the safety of applying photobiomodulation to the vaginal mucosa.

Conclusions or Pending Issues and List of Inadequacies: No inadequacies or pending issues were found and the rapporteur proposes that this research be considered APPROVED.

Once the research is completed, send the Final Report to the CEP in the form of a Notification, containing the summary of the work, informing the number of participants, conclusions and data on the publication status (sent, accepted or published).

Machine Translated by Google

Final Considerations at the discretion of the

CEP: The Board fully accepted the opinion issued by the reporting member.

This project was sent to CONEP for analysis on this date and its start will only be authorized after approval by the same.

This opinion was prepared based on the documents listed below:

Type Document File PB_INFORMAÇÕES_BÁSICAS_DO_P Basic		Post	Author	Situation
ProjetoFerrazACNrev.docx<br>Researcher Brochure TCLE / Terms of Consent / Justification for	Project Information ROJETO_2473758.pdf Detailed Project /	15/01/2025 16:48:00		Accepted
Absence Cover Page		15/01/2025 16:47:23	ANNA CAROLINA NUNES FERRAZ	Accepted
	TCLErev.docx	15/01/2025 16:46:38	ANNA CAROLINA NUNES FERRAZ	Accepted
	Facesheet.pdf	15/01/2025 16:43:18	ANNA CAROLINA NUNES FERRAZ	Accepted

Opinion Status:

Approved

Needs CONEP's Appreciation:

Yes

SAO PAULO, February 7, 2025

Signed by:
RENATO CARDOSO
(Coordinator)
