# Supplementary material for: Photobiomodulation for pain management during placement of the copper T 380 intrauterine device: Protocol for a randomized, double-blind controlled trial
Source: PLoS One. 2026 May 28;21(5):e0349031. doi: 10.1371/journal.pone.0349031 (PMC13218537; doi:10.1371/journal.pone.0349031)
Supplement: S6 File — This is the S6 File legend; there is no legend. (PDF) [file pone.0349031.s006.pdf]

ClinicalTrials.gov Protocol Registration and Results System (PRS) Receipt
Release Date: February 3, 2026

ClinicalTrials.gov ID: NCT06984796

Study Identification

Unique Protocol ID: 7.367.867

Brief Title: Photobiomodulation on Pain Perception During the Insertion of the T 380 Copper Intrauterine Device (IUD)

Official Title: Effect of Photobiomodulation on Reducing Pain Perception During the Insertion of the T 380 Copper Intrauterine Device (IUD) for Contraception: a Randomized Controlled Clinical Study

Secondary IDs:

Study Status

Record Verification: February 2026

Overall Status: Not yet recruiting

Study Start: March 30, 2026 [Anticipated]

Primary Completion: May 30, 2027 [Anticipated]

Study Completion: May 30, 2027 [Anticipated]

Sponsor/Collaborators

Sponsor: University of Nove de Julho

Responsible Party: Principal Investigator
Investigator: Anna Carolina Ratto Tempestini Horliana [AHorliana]
Official Title: Clinical Professor
Affiliation: University of Nove de Julho

Collaborators:

Oversight

U.S. FDA-regulated Drug: No

U.S. FDA-regulated Device: No

U.S. FDA IND/IDE: No

Human Subjects Review: Board Status: Approved
Approval Number: 7.367.867
Board Name: Complexo Hospitalar do Mandaqui
Board Affiliation: Complexo Hospitalar do Mandaqui
Phone: (11)2281-5147
Email: chm-cep@saude.sp.gov.br
Address:

Volunteers of the Nation Street, 4301 - Building 03 - Blue House
Santana
São Paulo - SP

Data Monitoring: Yes

Study Description

Brief Summary: Unplanned pregnancy affects up to 65% of women in some regions of Brazil, contributing to unsafe abortions and maternal mortality. The copper IUD is an effective long-term contraceptive but is underused, with only 4.4% of women of reproductive age using it. One barrier is the pain during insertion, leading to low adherence. Photobiomodulation (PBM), which has anti-inflammatory and analgesic effects, may offer a solution. This study aims to assess PBM's efficacy as a preemptive analgesic during copper IUD insertion in a randomized, double-blind trial involving 72 participants. The experimental group (n=36) will receive active PBM, while the control group (n=36) will receive PBM simulation. Pain will be measured using the Visual Analog Scale (VAS) at multiple time points, and additional outcomes include analgesic use, quality of life (WHOQOL-100), anxiety (GAD-7), satisfaction, and adverse effects. Statistical analysis will include tests such as the Friedman test, logistic regression, and ANOVA, with a significance level of 5%.

Detailed Description: Unplanned pregnancy affects up to 65% of women in some regions of Brazil, increasing the risks of unsafe abortions and contributing to maternal mortality. The copper IUD is an effective and long-lasting contraceptive option, but its use is still limited in Brazil, covering only 4.4% of women of reproductive age. One of the main barriers is the pain associated with its insertion, which leads to fear and low adherence to the method. Since pain can be of visceral or somatic origin, traditional approaches such as anti-inflammatories and anesthetics have shown inconclusive results in reducing this discomfort. Photobiomodulation (PBM) exhibits anti-inflammatory and analgesic effects and has demonstrated positive results in managing pelvic pain in various clinical contexts, including labor and delivery. The objective of this study is to evaluate the efficacy of PBM as a preemptive analgesic method during the insertion of the T 380 copper IUD. A randomized, double-blind clinical trial will be conducted with 72 participants randomly allocated into an experimental group (n=36) - active PBM and a control group (n=36) - PBM simulation. Patients will follow the IUD insertion protocol as outlined in the Ministry of Health guidelines. Pain will be assessed at different time points using the Visual Analog Scale (VAS) during the insertion phases (Pozzi, hysterometry, and IUD insertion), at 5 and 15 minutes, and at 24 and 48 hours after IUD insertion.

Additionally, analgesic use and quality of life (as measured by the WHOQOL-100) will be assessed over 48 hours, along with anxiety levels (as measured by the GAD-7), satisfaction with the procedure immediately after insertion (at 15 minutes), and adverse and side effects within 48 hours. The duration of pain, in hours, from the moment of IUD insertion until its resolution, will also be evaluated, as well as the success rate of the procedure. Statistical analysis will be performed using SPSS software version 24.0, with a significance level of 5% ($p < 0.05$). Data normality will be assessed using the Shapiro-Wilk test. Student's t-test or the Mann-Whitney test will be used for continuous variables, while the chi-square test or Fisher's exact test will be applied for categorical variables. Pain will be analyzed using the Friedman test, and logistic regression will be used to evaluate associations between groups and adverse effects. Statistical analysis will be performed with a significance level of 5%. Data normality will be assessed using the Shapiro-Wilk test.
For pain analysis (VAS) and variables such as anxiety and quality of life, the Friedman test will be applied. Analgesic use will be evaluated using a repeated

measures analysis of variance (ANOVA). Adverse effects will be analyzed using logistic regression. The time to resolve abdominal discomfort will be estimated using Kaplan-Meier analysis, and IUD insertion success will be compared using the chi-square test.

Conditions

Conditions: Pain

Keywords: intrauterine device
photobiomodulation

Study Design

Study Type: Interventional

Primary Purpose: Treatment

Study Phase: N/A

Interventional Study Model: Parallel Assignment

Number of Arms: 2

Masking: Triple (Participant, Investigator, Outcomes Assessor)

Allocation: Randomized

Enrollment: 72 [Anticipated]

Arms and Interventions

Arms	Assigned Interventions
Experimental: Photobiomodulation Group<br>All participants will undergo the same Intrauterine Device insertion procedure. For irradiation, a Light Emitting Diode panel from the brand Sportlux® (Brazil, SP) will be used, with the following specifications. Photobiomodulation in the experimental group will be administered using 132 Light Emitting Diodes, with 660 nm and 850 nm wavelengths, applied in contact mode. Each Light Emitting Diode has an emission area of 0.5 cm², with an application time of 20 minutes, an irradiance of 16 mW/cm², and an energy delivery of 4.8 J per Light Emitting Diode, resulting in a radiant exposure of 9.6 J/cm².	Device: Photobiomodulation<br>The irradiated region will cover the lumbar and thoracic spine, specifically from T10 to L4, using the Light Emitting Diode panel in a vertical orientation. Photobiomodulation in the experimental group will be administered using 132 Light Emitting Diodes with wavelengths of 660 nm and 850 nm, applied in contact mode. Each Light Emitting Diode has an emission area of 0.5 cm², with an application time of 20 minutes, an irradiance of 16 mW/cm², and an energy delivery of 4.8 J per Light Emitting Diode, resulting in a radiant exposure of 9.6 J/cm².<br>Other Names:<br>• low level laser therapy
Sham Comparator: Simulation of Photobiomodulation Group<br>All participants in this group will undergo the conventional Intrauterine Device insertion procedure, as previously described. They will receive a simulation of photobiomodulation and will be treated identically to the Experimental Group. The researcher responsible for the photobiomodulation application will simulate irradiation by positioning the device in the exact location as in the Experimental Group; however, the equipment will remain turned off.	Simulation of Photobiomodulation<br>The researcher responsible for the photobiomodulation application will simulate irradiation by positioning the device in the exact location as in the Photobiomodulation Group; however, the equipment will remain turned off. To prevent participants from identifying the group to which they belong, the device's activation sound (beep) will be pre-recorded and played during the application.

Arms	Assigned Interventions
To prevent participants from identifying the group to which they belong, the device's activation sound (beep) will be pre-recorded and played during the application.	

Outcome Measures

Primary Outcome Measure:

1. Pain in baseline
   VAS (Visual Analog Scale):

   Pain intensity was assessed using the Visual Analog Scale (VAS), a validated tool consisting of a 10-centimeter horizontal line ranging from 0 (no pain) to 10 (worst possible pain). Participants were instructed to mark a point on the line that best represented their perceived pain at each procedural step.

   [Time Frame: baseline]

2. Pain in five minutes
   VAS (Visual Analog Scale):

   Pain intensity was assessed using the Visual Analog Scale (VAS), a validated tool consisting of a 10-centimeter horizontal line ranging from 0 (no pain) to 10 (worst possible pain). Participants were instructed to mark a point on the line that best represented their perceived pain at each procedural step.

   [Time Frame: 5 minutes]

Secondary Outcome Measure:

3. Pain in Pozzi Clamp Placement
   VAS (Visual Analog Scale):

   Pain intensity was assessed using the Visual Analog Scale (VAS), a validated tool consisting of a 10-centimeter horizontal line ranging from 0 (no pain) to 10 (worst possible pain). Participants were instructed to mark a point on the line that best represented their perceived pain at each procedural step:

   Pozzi Clamp Placement: A tenaculum is gently applied to the anterior lip of the cervix to align the uterine axis and provide cervical stabilization, facilitating safe access to the uterine cavity.

   [Time Frame: Immediately at the time of Pozzi clamp placement, assessed within 1 minute of application]

4. Pain in Hysterometry
   VAS (Visual Analog Scale):

   Pain intensity was assessed using the Visual Analog Scale (VAS), a validated tool consisting of a 10-centimeter horizontal line ranging from 0 (no pain) to 10 (worst possible pain). Participants were instructed to mark a point on the line that best represented their perceived pain at each procedural step:

   Hysterometry: A sterile uterine sound is inserted to assess the direction and depth of the uterine cavity, which is essential for guiding correct IUD placement and minimizing complications.

   [Time Frame: Immediately at the time of hysterometry, assessed within 1 minute of the procedure]

5. Pain in Intrauterine Device Insertion
   VAS (Visual Analog Scale):

   Pain intensity was assessed using the Visual Analog Scale (VAS), a validated tool consisting of a 10-centimeter horizontal line ranging from 0 (no pain) to 10 (worst possible pain). Participants were instructed to mark a point on the line that best represented their perceived pain at each procedural step:

   Intrauterine Device Insertion: The IUD is carefully introduced through the cervical canal and positioned at the uterine fundus. Once released, the device remains in place, and the strings are trimmed to ensure proper follow-up and future removal.

   [Time Frame: Immediately at the time of IUD insertion, assessed within 1 minute of the procedure]

6. Pain in 15 minutes
VAS (Visual Analog Scale):

Pain intensity was assessed using the Visual Analog Scale (VAS), a validated tool consisting of a 10-centimeter horizontal line ranging from 0 (no pain) to 10 (worst possible pain). Participants were instructed to mark a point on the line that best represented their perceived pain at each procedural step.

[Time Frame: 15 minutes]

7. Pain in 24 hours
VAS (Visual Analog Scale):

Pain intensity was assessed using the Visual Analog Scale (VAS), a validated tool consisting of a 10-centimeter horizontal line ranging from 0 (no pain) to 10 (worst possible pain). Participants were instructed to mark a point on the line that best represented their perceived pain at each procedural step.

[Time Frame: 24 hour]

8. Pain in 48 hours
VAS (Visual Analog Scale):

Pain intensity was assessed using the Visual Analog Scale (VAS), a validated tool consisting of a 10-centimeter horizontal line ranging from 0 (no pain) to 10 (worst possible pain). Participants were instructed to mark a point on the line that best represented their perceived pain at each procedural step.

[Time Frame: 48 hour]

9. Number of analgesics taken in baseline
Paracetamol intake was recorded at the T 380 copper IUD insertion baseline. At the beginning of the study, each participant will receive a blister pack of paracetamol (a pure analgesic) (Jóźwiak-Bebenista, 2014), which must be kept until the end of the study and will only be used in cases of pain. At the end of the study, the remaining number of tablets will be assessed as an additional pain measurement parameter. The analgesic used will be paracetamol 500 mg, to be taken only if necessary at a dose of one tablet every six hours. Participants will be asked to record in writing the number of tablets taken, along with the date and time of consumption. A monitoring procedure will be implemented to ensure adherence: participants must bring their blister pack to follow-up visits for verification.

[Time Frame: Baseline]

10. Number of analgesics taken in a total period of 48 hours
Paracetamol intake was recorded from baseline to 24 hours after the T 380 copper IUD insertion. At the beginning of the study, each participant will receive a blister pack of paracetamol (a pure analgesic), which must be kept until the end of the study and will only be used in pain cases. At the end of the study, the remaining number of tablets will be assessed as an additional pain measurement parameter. The analgesic used will be paracetamol 500 mg, to be taken only if necessary, at a dose of one tablet every six hours. Participants will be asked to record in writing the number of tablets taken, along with the date and time of consumption. A monitoring procedure will be implemented to ensure adherence: participants must bring their blister pack to follow-up visits for verification. Medication use will be tracked from baseline to 24 hours post-insertion.

[Time Frame: 48 hours]

11. Anxiety assessment in baseline
Generalized anxiety symptoms were evaluated at baseline in patients using the T 380 copper intrauterine device for contraception, through the Generalized Anxiety Disorder 7-item (GAD-7) questionnaire. This self-administered tool, based on DSM-IV criteria, assesses the frequency of anxiety symptoms over the prior two weeks using a four-point Likert scale: 0 (never), 1 (several days), 2 (more than half the days), and 3 (nearly every day). Suitable for clinical and research use, and applicable in person or digitally, it takes about five minutes to complete. Total scores range from 0 to 21, with higher scores indicating more severe symptoms, categorized as follows: 0–4 (no anxiety), 5–9 (mild), 10–14 (moderate), and 15–21 (severe anxiety).

[Time Frame: at Baseline]

12. Anxiety assessment after 15 minutes of insertion
Generalized anxiety symptoms were evaluated at baseline in patients using the T 380 copper intrauterine device for contraception, through the Generalized Anxiety Disorder 7-item (GAD-7) questionnaire. This self-administered tool, based on DSM-IV criteria, assesses the frequency of anxiety symptoms over the prior two weeks using a four-point Likert scale: 0 (never), 1 (several days), 2 (more than half the days), and 3 (nearly every day). Suitable for clinical and

research use, and applicable in person or digitally, it takes about five minutes to complete. Total scores range from 0 to 21, with higher scores indicating more severe symptoms, categorized as follows: 0–4 (no anxiety), 5–9 (mild), 10–14 (moderate), and 15–21 (severe anxiety).

[Time Frame: 15 minutes after the insertion]

13. Quality of life assessment at baseline
Measured using the WHOQOL-Pain instrument at Baseline. The WHOQOL-Pain is an additional module of the WHOQOL-100, specifically developed to assess quality of life in individuals experiencing chronic pain. Each facet consists of four questions addressing different dimensions of the pain experience. Responses follow a five-point Likert scale, measuring intensity, capacity, evaluation, and frequency. Scores are converted to a 0 to 100 scale, allowing for a quantitative interpretation of pain levels and their impact on quality of life. The WHOQOL-Pain items are not integrated into the WHOQOL-100 and are applied separately at the end of the main questionnaire. This structure facilitates individualized analysis of pain-related facets, enabling a specific and detailed evaluation of physical chronic pain experiences.

[Time Frame: at baseline]

14. Quality of life assessment after 48 hours
Measured using the WHOQOL-Pain instrument from baseline to 48 hours after insertion. The WHOQOL-Pain is an additional module of the WHOQOL-100, specifically developed to assess quality of life in individuals experiencing chronic pain. Each facet consists of four questions addressing different dimensions of the pain experience. Responses follow a five-point Likert scale, measuring intensity, capacity, evaluation, and frequency. Scores are converted to a 0 to 100 scale, allowing for a quantitative interpretation of pain levels and their impact on quality of life. The WHOQOL-Pain items are not integrated into the WHOQOL-100 and are applied separately at the end of the main questionnaire. This structure facilitates individualized analysis of pain-related facets, enabling a specific and detailed evaluation of physical chronic pain experiences.

[Time Frame: 48 hours after the insertion]

15. Participant Satisfaction
Patient satisfaction with T 380 copper intrauterine device insertion for contraceptive purposes was assessed 15 minutes after the procedure using a dichotomous questionnaire adapted from Lopes et al. (2015). The instrument included three yes-or-no questions: (1) Was the insertion experience pleasant? (2) Would you undergo the procedure again in the future? (3) Would you recommend it to a friend? For each item, the response "yes" indicated satisfaction with the procedure, while "no" reflected dissatisfaction. Responses were scored as 1 for "yes" and 0 for "no", resulting in a total score ranging from 0 (maximum satisfaction) to 3 (maximum dissatisfaction). Higher scores indicated greater dissatisfaction with the procedure.

[Time Frame: 15 minutes after insertion]

16. Time required for abdominal discomfort relief (menstrual cramps)
The time required to relieve abdominal discomfort (menstrual cramps) was recorded from the day of the baseline assessment.

[Time Frame: baseline]

17. Time required for abdominal discomfort relief (menstrual cramps)
The time required to relieve abdominal discomfort (menstrual cramps) was recorded until the day the discomfort stopped. The patient will be asked after 48 h

[Time Frame: 48 hours after insertion]

18. Successful insertion
If IUD insertion fails, it will be assessed dichotomously (success/failure) from baseline to 24 hours after insertion, and verified by ultrasonography when necessary.

[Time Frame: 48 hours after insertion]

19. Adverse effects
Including uterine perforation, IUD displacement, abdominal pain, increased vaginal bleeding, and allergic reactions. An open-ended question will allow participants to report adverse effects, followed by a list of potential effects to help recall any unreported symptoms.

[Time Frame: 48 hours]

20. Side effects

Including cramps, mild pain, light bleeding, and tongue numbness. An open-ended question will be asked first, followed by a list of potential side effects for better recall.

[Time Frame: 48 hours]

Eligibility

Minimum Age: 18 Years

Maximum Age: 50 Years

Sex: Female

Gender Based:

Accepts Healthy Volunteers: Yes

Criteria: Inclusion Criteria:

- Women aged 18–50 years,
- Female,
- No preference regarding race or socioeconomic status,
- Nulliparous or multiparous.

Exclusion Criteria:

- Known or suspected pregnancy,
- Diagnosed with chronic pain,
- Active local infection,
- Use of any pain medication in the last 12 hours,
- Known contraindication for IUD insertion, including significant uterine cavity distortion, active pelvic inflammatory disease, or Wilson's disease,
- Allergy to copper,
- Unexplained abnormal uterine bleeding,
- Any condition affecting the lumbar region, such as active neoplasms, established osteomyelitis, or any pre-existing deep tissue lesions with necrosis or infection,
- History of photosensitivity.

Contacts/Locations

Central Contact Person: ANNA CAROLINA N FERRAZ, PhD student
Telephone: 11996324513
Email: Anna Carolina Nunes Ferraz <annacarolina1984@gmail.com>

Central Contact Backup: Anna Carolina Ratto Tempestini Horliana, PhD
Telephone: 11948002288
Email: annacrth@gmail.com

Study Officials: Anna Carolina Nunes Ferraz
Study Principal Investigator
University of Nove de Julho

Locations: **Brazil**
Nove de Julho University (UNINOVE)
São Paulo, Brazil, 01504-001
Contact: Anna Carolina RT Horliana, PhD +5511 3385-9197
annacrth@gmail.com

IPDSharing

Plan to Share IPD:

References

Citations:

Links:

Available IPD/Information:

U.S. National Library of Medicine | U.S. National Institutes of Health | U.S. Department of Health & Human Services
